# *De Novo* Transcriptomes of *Forsythia koreana* Using a Novel Assembly Method: Insight into Tissue- and Species-Specific Expression of Lignan Biosynthesis-Related Gene

**DOI:** 10.1371/journal.pone.0164805

**Published:** 2016-10-21

**Authors:** Akira Shiraishi, Jun Murata, Erika Matsumoto, Shin Matsubara, Eiichiro Ono, Honoo Satake

**Affiliations:** 1 Bioorganic Research Institute, Suntory Foundation for Life Sciences, Kyoto, 619-0284, Japan; 2 Research Institute, Suntory Global Innovation Center (SIC) Ltd., Kyoto, 619-0284, Japan; Jawaharlal Nehru University, INDIA

## Abstract

*Forsythia* spp. are perennial woody plants which are one of the most extensively used medicinal sources of Chinese medicines and functional diets owing to their lignan contents. Lignans have received widespread attention as leading compounds in the development of antitumor drugs and healthy diets for reducing the risks of lifestyle-related diseases. However, the molecular basis of *Forsythia* has yet to be established. In this study, we have verified *de novo* deep transcriptome of *Forsythia koreana* leaf and callus using the Illumina HiSeq 1500 platform. A total of 89 million reads were assembled into 116,824 contigs using Trinity, and 1,576 of the contigs displayed the sequence similarity to the enzymes responsible for plant specialized metabolism including lignan biosynthesis. Notably, gene ontology (GO) analysis indicated the remarkable enrichment of lignan-biosynthetic enzyme genes in the callus transcriptome. Nevertheless, precise annotation and molecular phylogenetic analyses were hindered by partial sequences of open reading frames (ORFs) of the Trinity-based contigs. To obtain more numerous contigs harboring a full-length ORF, we developed a novel overlapping layout consensus-based procedure, virtual primer-based sequence reassembly (VP-seq). VP-seq elucidated 709 full-length ORFs, whereas only 146 full-length ORFs were assembled by Trinity. The comparison of expression profiles of leaf and callus using VP-seq-based full-length ORFs revealed 50-fold upregulation of secoisolariciresinol dehydrogenase (SIRD) in callus. Expression and phylogenetic cluster analyses predicted candidates for matairesinol-glucosylating enzymes. We also performed VP-seq analysis of lignan-biosynthetic enzyme genes in the transcriptome data of other lignan-rich plants, *Linum flavum*, *Linum usitatissimum* and *Podophyllum hexandrum*. The comparative analysis indicated both common gene clusters involved in biosynthesis upstream of matairesinol such as SIRD and plant lineage-specific gene clusters, in particular, genes responsible for biosynthetic pathways for production of podophyllotoxin; CYP71BE54, a key enzyme gene for podophyllotoxin biosynthesis in *P*. *hexandrum*, was not found in *L*. *flavum*, although both *P*. *hexandrum*. and *L*. *flavum* yield podophyllotoxin. Altogether, these data have established the fruitful molecular basis of *Forsythia* and provided insight into the molecular evolution and diversity of lignan biosynthetic pathways.

## Introduction

Plants biosynthesize a wide variety of specialized metabolites (formerly termed secondary metabolites), including alkaloids, flavonoids, isoflavonoids, and lignans [1–3]. These phytochemicals are produced by plant species-specific enzymatic biosynthesis cascades that are regulated by multiple endogenous factors and exogenous stimuli at genomic and transcriptional levels. Many plant specialized metabolites serve as major bioactive components in functional diets and clinical agents, including Chinese medicines, and as leading compounds in the development of novel synthetic drugs. The considerable recent increases in the aging population require the improvement of a healthy life expectancy via consistent and appropriate intake of low-cost healthy diets and clinical drugs, many of which are derived from plants. Furthermore, little is known about the biological significance of plant specialized metabolites in their host plants. Thus, comprehensive investigation, including transcriptomic analysis, of the regulatory mechanisms of plant specialized metabolite biosynthesis and associated biological processes is of particular importance in light of both medicinal and plant science.

*Forsythia* spp. (Oleaceae family), commonly known as Golden Bells, are perennial woody plants that include a large number of natural and cultivated varieties. The leaves and fruits are one of the most extensively used medicinal sources of Chinese medicines and functional diets owing to their high lignan contents [1–3]. Over the past few decades, the lignan biosynthetic pathways of *Forsythia* have been identified [1–5]. There is a growing body of evidence for the diverse biological effects of lignans on mammals, including antioxidative, antiobesity, antitumor, antiviral activities, and the reduction of the risk of various cancers and cardiovascular diseases [6–8]. Quite recently, we also demonstrated the marked usefulness of triple-transgenic *Forsythia* callus suspensions as efficient, stable, and sustainable platforms for the production of a beneficial non-*Forsythia* lignan, sesamin [9].

Collectively, these findings highlighted the potentially high significance of transcriptomic information of lignan-rich plants in more efficient transgenic metabolic engineering of lignan biosynthesis pathways as well as phytochemical studies of lignan biosynthesis. However, such molecular basis of *Forsythia* remains to be explored. A next-generation sequencing-based transcriptome is expected to provide a vast amount of fundamental information as to genes of enzymes responsible for unidentified lignan biosynthesis steps and/or gene expression profiles associated with lignan biosynthesis. In this paper, we investigated the *de novo* transcriptomes of *Forsythia koreana* leaf and callus using a novel contig assembly method, and compared the gene expression profiles of *Forsythia* with those of other lignan-rich plants: *Podophyllum hexandrum*, *Linum usitatissimum*, and *L*. *flavum*.

## Materials and Methods

### Plant material and RNA extraction

Calli were induced from the developing first leaf of *F*. *koreana* plants grown in vitro under germ-free conditions. Subsequently, cell suspension culture lines of *F*. *koreana* were established from the calli as described previously [9] and were maintained at 22°C in Gamborg’s B-5 liquid medium supplemented with 6% sucrose and 0.05 mg l-1 2,4-D (Cell Culture Medium; CCM). All suspensions were cultured on a rotary shaker at 110 rpm in the dark and were subcultured every 2 weeks with an inoculum of 5 ml of saturated suspension cells. Cell suspension cultures that have been maintained for 3 months were used for RNA extraction. Total RNA was extracted from *in vitro*-grown leaf or callus suspension cultures that had been maintained for 3 months, using an RNeasy Plant Mini Kit (Qiagen, Hilden, Germany) and was subjected to on-column DNase I digestion according to the manufacturer’s instruction.

### RNA-Seq and assembly

The cDNA libraries for RNA-seq were constructed using the TruSeq Stranded mRNA Sample Prep Kit (Illumina, Inc., San Diego, CA, USA), according to the manufacturer’s protocol. The quality and concentrations of the libraries were verified using a Qubit fluorometer (Thermo Fisher Scientific, Waltham, MA, USA) and a BioAnalyzer system (Agilent Technologies, Santa Clara, CA, USA), as previously reported [10]. The libraries were then subjected to 101 × 2 cycles of paired-end sequencing, using the HiSeq 1500 platform (Illumina) in High Output Mode.

Total reads were extracted using CASAVA v1.8.2 (Illumina), and subsequently, PCR duplicates, adaptor sequences, and low-quality reads were removed from the extracted reads as follows. Briefly, if the first 10 bases of the two reads were identical and the entire reads exhibited >90% similarity, the reads were considered PCR duplicates. Base calling from the 5′ to the 3′ end was started when the Phred scores of five sequential residues exceeded 30 and stopped when the frequency of accurately called bases dropped to 0.5. The remaining reads were assembled using Trinity (v2.0.6) [11], with normalization and a maximum coverage of 30. For each sample, the reads were aligned using a two-step method, in order to obtain reliable fragment per kilobase of transcript per million mapped reads (FPKM) values. Putative gene names for the contigs were estimated based on a homology search of the UniProt database, using blastn 2.29+ [12]. To determine expression levels, the total numbers of mapped reads were estimated using Bowtie (v2.1.0) [13], and the short reads were mapped against the contigs with Bowtie, allowing single mutations and ignoring the mismatch penalty for nucleotides with low-quality values (<20).

### Gene ontology (GO) analysis and identification of plant specialized metabolism -related contigs

To predict the function of leaf- and callus-specific transcripts, the specific sequences were analyzed using GO enrichment analysis, as described previously [10]. Briefly, specific or constitutively expressed sequences were analyzed using Blast2GO for functional annotation [14], and the annotated biological information was then compared using GO ‘is_a’ graphs. The graph was drawn with Cytoscape (http://www.cytoscape.org/). To quantify the enrichment of GO terms, we calculated enrichment scores as follows:

Enrich(GO)=log2Nleaf<<callus(GO)NTotal(leaf<<callus)Ncallus<<leaf(GO)NTotal(callus<<leaf) (1)

where *N*_*x*_*(GO)* indicates the frequency of each GO term for leaf (leaf<<callus)- or callus (callus<<leaf)-specific genes (i.e., *x*), and *N*_*total*_*(X)* indicates the frequency of leaf (leaf<<callus)- or callus (callus<<leaf)-specific genes (i.e., *X*) mapped to each GO term in the Blast2GO results. For correction, a pseudo-count was set as fixed value (0.05).

Contigs with sequences similar to enzymes involved in special metabolites biosynthesis were identified using blastx against a local reference database of Swiss-Prot data with GO terms for secondary metabolic processes (GO:0019748), with an e-value threshold of 0.001. Full-length ORFs were detected using the algorism of TargetIdentifier [15].

### Generation of full-length ORF sequences of contigs using Virtual Primer-Based method

**Fig 1:**
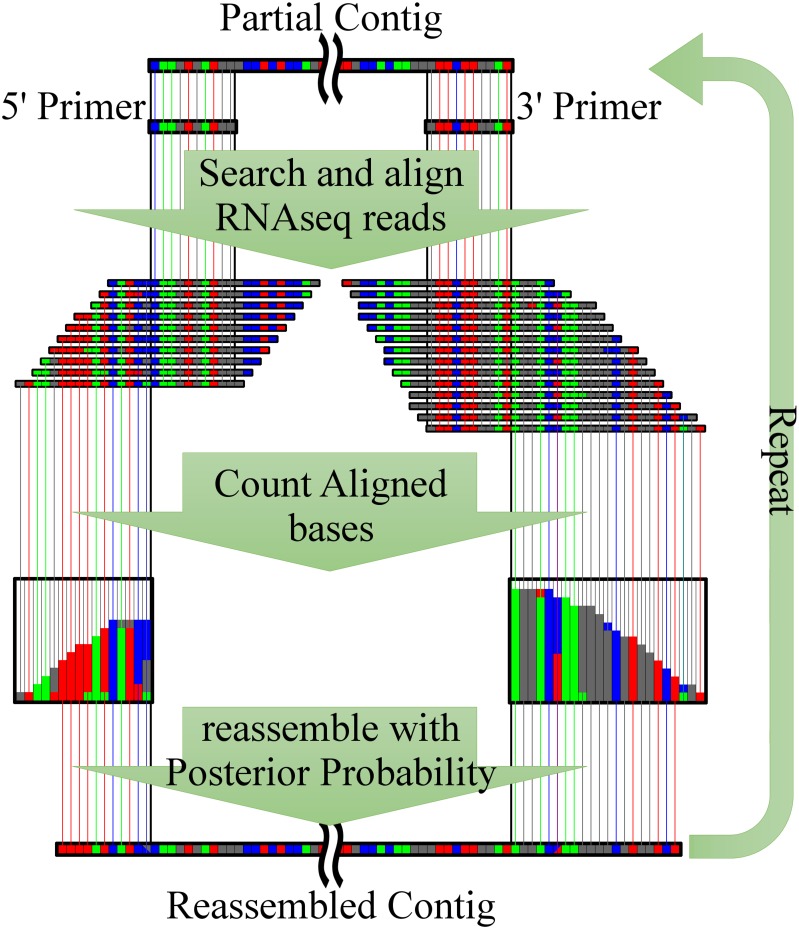
Schematic of virtual primer-based sequence assembly (VP-seq). Short reads that contain terminus regions of incomplete open reading frame (ORF) sequences are collected and aligned. The contigs containing the incomplete ORFs are then reassembled with posterior probabilities, which are calculated from the aligned short reads. The process is repeated until no further significant elongation is found at either terminus.

Trinity employs the de Bruijn graph algorism by splitting reads into much shorter k-mers, in order to efficiently handle the massive number of reads within a practical length of time. However, the overlap-layout-consensus assembly algorism [16, 17] is more suitable for regions of low-depth coverage. Therefore, to overcome the difficulties caused by the low-depth regions of the Trinity-based contigs and to generate full-length ORF sequences, we developed a memory and time-efficient overlap-layout-consensus reassembling method, virtual primer-based sequence assembly (VP-seq; Fig 1), which comprises the following steps: (1) reads that were not mapped to Trinity-based contigs and reads mapped to transcripts were collected and stored in a hash table without pairing information; (2) the search key (virtual primer) sequences with a length of 20 bp were set for the 5′ and 3′ ends of the transcripts; (3) reads containing the primer sequences were mapped to the incomplete ORF sequences; and (4) the elongated nucleotide sequences were determined from the primers (5′ primer to 5′ end and 3′ primer to 3′ end), using the following equation:

$$nt\left(i\right)=\{\begin{array}{ll} \underset{nt=ATGC}{\text{arg max}}\text{ Pr}\left(nt,i\right), & \underset{nt=ATGC}{\text{max}}\text{Pr}\left(nt,i\right)\geq 0.8\\ \underset{nt=RYKMSW}{\text{arg max}}\text{Pr}\left(nt,i\right), & \underset{nt=RYKMSW}{\text{max}}\text{ Pr}\left(nt,i\right)\geq 0.8\cap nt\left(i-1\right)=\left(A,T,G,C\right)\\ no\;elongation, & otherwise \end{array},$$ (2)

where the posterior probabilities of nucleotides were calculated and the elongating nucleotides including ambiguous nucleotides (R, Y, K, M, S, W) were selected to maximize the posterior probabilities, and to avoid low complexity or sequence contamination, the occurrence of successive ambiguous nucleotides was limited. Furthermore,

$$\text{Pr}\left(nt,i\right)=\frac{\frac{N\left(nt|position=i\right)+n/4}{N\left(position=i\right)+n}}{1/4},$$ (3)

where *N(position = i)* represents the number of reads mapped to position *i* and *N(nt | position = i)* is the number of reads with contained nucleotide *nt* at position *i*. For correction, a pseudo-count *n* was set to 20, and finally, (5) the process was repeated from step (2) and until no nucleotides were added to either end. Full-length ORFs were detected using the algorism of TargetIdentifier [15].

Unlike the OLC algorism, VP-seq utilizes only both ends of contigs as virtual primer regions to reduce computational cost. To collect adequate reads with virtual primer-based search for posterior probability calculation and contig elongation, two features were implemented in VP-seq. Firstly, a single mismatch was allowed for searching reads containing virtual primer sequences. In the first-round VP-seq, which utilized virtual primer regions corresponding to both ends of original contigs obtained using Trinity, a single mismatch was allowed between the first virtual primers and reads containing the virtual primer sequences. This mismatch allowance enabled the complimentary recognition of read sequences containing a single base call error or a single nucleotide variation by virtual primers, followed by the posterior probability estimation and elongation. Secondly, the first-round VP-seq-based contigs containing ambiguity nucleotide codes were used as templates for design of the second- or later-round virtual primers for further contig elongation. Consequently, contigs were further elongated using read sequences derived from both heterogenous alleles regardless of nucleotide variations at virtual primer regions. To evaluate the efficiency of VP-seq, the computation time for VP-seq including the process of Trinity and TGICL was monitored.

### Quantitative reverse transcription-PCR (qRT-PCR)

To verify the results obtained by RNA-Seq, the expression of three lignan biosynthetic genes (*FkSIRD*, *FkMOMT*, and *FkUGT74S1*) was analyzed by qRT—PCR as previously described [18] using the following primer sets: FkSIRD-FW (5'- CCTAT TTATG AAGCA CGCAG CAC -3') and FkSIRD-RV (5'- AGGAA GCCCG AAAGG AGACA -3') for *FkSIRD*; FkMOMT-FW (5'-GCAAG GGTGG CAATG TCAGC-3') and FkMOMT-RV (5'-ATCAC CACCT CCAAC ATCCA C-3') for *FkMOMT* and FkUGT74S1-FW (5'- GAATC CGAGC GTCAT CAGA -3') and FkUGT74S1-RV (5'- GTGGC ATTTT TCTTC ATCTC TTCC -3') for *FkUGT74S1*. The *F*. *koreana* EF1A gene expression was used as an internal control, using primers: EF1A-FW (5'- CATGG TTGTT CAATT GCAAG GGAGC -3') and EF1A-RV (5'- CGCCT GTCAA GTACC CAATT C -3').

### Phylogenetic analysis for lignan-biosynthetic enzymes

The RNA-seq data for VP-seq and FPKM calculation of *P*. *hexandrum*, *L*. *usitatissimum*, and *L*. *flavum* were obtained from the MedPlant RNA Seq Database (http://www.medplantrnaseq.org/; assembled contigs version 20101112). After eliminating sequences without Pfam domains, the full-length sequences obtained *via* VP-seq were translated and subjected to Pfam search [18]. The hit sequences for the respective Pfam domains were then translated to amino acid sequences, aligned using ClustalW2, and phylogenetic trees were reconstructed using unweighted pair group method with arithmetic mean (UPGMA) in ClustalW2.

## Results and Discussion

### RNA-Seq and *de novo* assembly

The RNA-Seq for the *F*. *koreana* callus and leaf using HiSeq 1500 yielded 41 and 47 million reads for 101 paired-end read, respectively (Table 1). The resulting fastq files were deposited on SRR2075825 (callus) and SRR2075824 (leaf). After base calling for clean reads, we obtained 39 and 45 million reads, respectively, with the average length of 97.4 bp paired-end reads (Table 1). Trinity (v2.0.6) analysis of the paired-end reads generated 383,714 contigs. Furthermore, reassembly of these contigs using TGICL (v2.1) led to the elucidation of 116,825 unigenes with an N50 of 977 from both the callus and leaf (Table 1). The unigenes covered 86.16% and 85.31% of reads from the callus and leaf libraries, respectively, and 79.46% of the unigenes were mapped *via* blastx to known sequences in the NBI nr database, confirming quality values comparable to those of other *de novo* RNA-seq results [19]. Subsequently, we compared the gene expression levels between the leaf and callus. 83.34% (97,055) of the unigenes yielded FPKM values of >0.1. Of those, 3963 genes and 2880 genes were upregulated in the leaf and calls with log ratios of >2 or <-2.

**Table 1:** Metrics of the *de novo* assembly of the *Forsythia koreana* transcriptome.

Sample	Read length	# reads	# contigs	N50 (bp)	Mean length (bp)	# PSM enzyme-related sequences
Leaf	101 bp × 2	41,267,276	116.824	977	741.0	1,576
Callus	101 bp × 2	47,841,039				

PSM, plant specialized metabolite.

### GO term enrichment analysis

**Fig 2:**
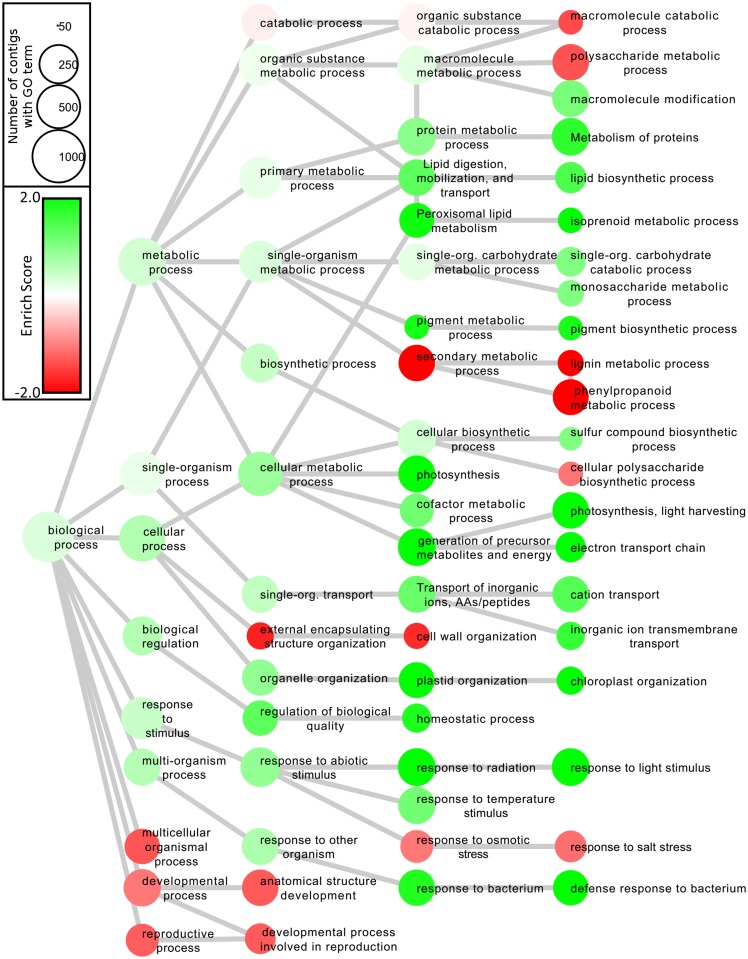
Gene Ontology (GO) enrichment analysis of differentially expressed genes in *Forsythia* leaf and callus. Each node represents a GO term. The branches of GO hierarchical trees without significantly enriched GO terms are not shown. The size and color of each node indicate the number of contigs with the GO terms and the enrichment score, respectively. Edges represent ‘is_a’ connections between GO terms.

To predict the biological functions of the 6,843 genes differentially expressed in the leaf and callus (3,963 genes in the leaf, and 2,880 genes in the callus), the resulting contig sequences were annotated by BLAST2GO [14]. The enrichment score was calculated as described in our previous report [10]. The annotated biological information between leaf and callus was compared by heat map in graph view for biological process (Fig 2). Nodes represent ontology terms and edges are 'is_a' relations used in the GO Biological Process ontology.

As depicted in Fig 2, photosynthesis-related (photosynthesis, light harvesting: GO:0009765; electron transport chain: GO:0022900; chloroplast organization: GO:0009658; response to light stimulus: GO:0009416); inorganic ion, amino acid, and peptide transport-related (GO:0006811); protein metabolic process-related (GO:0019538); and lipid metabolism-related (GO:0008610 and GO:0006720) genes were upregulated in the *F*. *koreana leaf*, whereas metabolic process-related (secondary metabolic process: GO:0019748; macromolecule catabolic process: GO:0009057), body structure organization-related (external encapsulating structure organization: GO:0045229), anatomical structure development-related (GO:0048856 and GO:0003006), and salt stress response-related (GO:0009651) genes were upregulated in *F*. *koreana* callus.

More than 50% of upregulated transporter-related (GO:0006811; 27/48) and protein metabolic process-related genes (GO:0019538; 67/121) were chloroplast proteins responsible for photosynthesis and electron transport. For example, the photosystem I complex subunits psaK (*TR52663_c0_g1_i1*), psiOs (*TR52663_c0_g1_i1* and *TR48474_c0_g2_i1*), and petC (CL19679Contig1), which constitute an integral chloroplast membrane protein complex for photosynthetic light reactions, were all upregulated in leaf and categorized as both photosynthesis- and transport-related processes. Moreover, the upregulated protein metabolic process genes reflected the rapid degradation and reassembly cycle of the photosystem complex. In addition, the chlorophyll-binding proteins LHCA (*CL1468Contig3*, *CL3961Contig1*, *CL12344Contig1*, and *CL15018Contig1*) and LHCB (*TR48065_c1_g3_i4*, *CL427Contig2*, *CL8878Contig1*, and *TR45565_c1_g2_i1*), as well as FtsH1 (*CL11358Contig1*), which is a component of the FtsH protease, were all upregulated in leaf and were categorized as both photosynthesis and protein metabolic processes. In addition, the genes for lipid metabolic process were also upregulated in the leaf. These GO terms were typical biological processes in leaves, where photosynthetic-related metabolic processes are exerted under light condition.

In contrast, the anatomical structure development-related (GO:0048856) and salt stress response-related (GO:0009651) genes were upregulated in callus. Such gene upregulation was also detected in *Arabidopsis thaliana* calli [20]. Intriguingly, the most enriched GO term in the callus was found to be implicated with secondary metabolic process (GO:0019748). Therefore, we investigated the functional characteristics of *Forsythia* genes involved in specialized metabolite synthesis.

### Collection and annotation of plant specialized metabolism -related enzymes using “Virtual Primer”-based sequence assembly

Considering that *Forsythia* is a well-known medicinal plant that produces a wide variety of phenylpropanoid-derived specialized metabolites including lignans, we attempted to annotate the sequences of these metabolite-related genes. Initially, we subjected the contigs to blastx analysis against known UniProt enzyme sequences with each Pfam motif (Table 2), and obtained 1,576 contigs with e-values of <0.001. However, 90.8% (1,430) of the contigs harbored partial ORFs and no Pfam domain, which were insufficient for precise functional annotation and molecular phylogenetic analyses. Such incomplete annotation of partial ORFs obtained using Trinity was frequently found in other Trinity-based contigs of *de novo* transcriptomes [21, 22], indicating that most of Trinity contigs can be applied for GO enrichment, but not for precise annotation and molecular phylogenetic analyses. Such short contigs are attributed to the Trinity’s k-mer-based fractionation methods, given that de Bruijn graph-based algorisms, including Trinity, fractionate the reads to k-mers and select k-mers (>1.5 information contents) to reduce the calculation cost. This fractionation can be optimized only for highly expressed regions, resulting in the formation of short contigs [23]. Such strategy is an efficient computation approach for assembling vast amounts of short reads and is adopted by most *de novo* RNA assemblers, such as SOAPdenovo-Trans [24], Velvet/Oases [25] and Trans-ABySS [26]. However, the k-mer size cannot be optimized for whole transcript sequences due to the heterogeneous depth caused by different expression levels of respective transcripts and the exclusion of read number information by k-mer selection.

**Table 2:** Representative protein families involved in plant specialized metabolisms.

Name	Example	Accession	Pfam Code
NADPH-dependent PLR, IFR,and PCBER (PIP) family reductase	Fi_pinoresinol larisiresinol reductase	U81158	NmrA
NAD-dependent dehydrogenase	Fi_secoisolariciresinol dehydrogenase	Q94KL7	adh_short
cytochrome P450 monooxygenase	Si_CYP81Q1	AB194714	p450
	Sh_CYP71CU	KT390172	
	Sh_CYP719A24	AGC29954	
	Sh_CYP71B56	KT390179	
	Sh_CYP82D61	KC110995	
2-oxoglutrate dependent dioxygenase	Sh_2ODD	KT390173	DIOX_N
UDP-sugar dependent glucosyltransferase	Si_UGT71A9	AB293960	UDPGT
	Si_UGT94D1	AB333799	
	Lu_UGT74S1	AGD95005	
SAM dependent O-methyltransferase	Sh_OMT3	KT390157	Methyltransf_2
			Methyltransf_3
	Sh_OMT1	KT390155	
			Methyltransf_11
Polyphenol oxidase	Lt_Larreatricin hydroxylase	AAQ67412	PPO1_DWL
Acyltransferase	Cs_cucurbitacin C acetyltransferase	KM655854	Transferase
Terpene cyclase/Terpene synthase	Sr_copalyl diphosphate synthase	AER58181	
	Sr_Kaurene synthase	AAD34295	
Polyketide Synthase	In_Chalcone synthase	BAA87336	Chal_sti_synt_N
			Polyketide_cyc2
alcohol/aldehyde dehydrogenase	Aa_ADH1	AEI16475	ADH_zinc_N
	AaALDH1	ACR61719	ADH_N

To increase contigs containing full-length ORFs, we developed a post-assembly method, named Virtual Primer-based Sequence assembly (VP-seq) that elongates 5’- and 3’-ends of generated contigs. VP-seq involves reassembly of Trinity-based contigs using an overlap-layout-consensus (OLC) algorism [16, 17] that does not fractionate reads to k-mers. Eventually, Trinity-based contigs are elongated *in silico* which is reminiscent of rapid amplification of cDNA ends (RACE).

The OLC algorism involves all-against-all and pairwise read alignments. Although many OLC algorism-based assemblers including Newbler and Celera assembler apply for K-mer based seed & extend algorism to find overlapped sequences to reduce the calculation cost, pairwise alignment step still has O(n^2^) computational cost, where O(*x*) represents the cost for calculation directly proportional to *x* and n is the number of input reads. Indeed, Newbler failed to complete the pairwise alignment step for 89 million *Forsythia* paired end RNA seq reads in one month. On the other hand, the computational order for VP-seq is O(n*m), where n is the number of input reads and m is the number of contigs of interest from Trinity outputs, which is much smaller than n, given that VP-seq involves all-against-target and pairwise alignments for virtual primer sequence-containing reads. Indeed, VP-seq, including Trinity and TGICL (32 hours), estimated the full length output for 1,576 specialized metabolism-related contigs in 62 hours using a 80-core 2TB server for our following validation. These results indicated the marked advantages in efficient calculation cost and precise elongation of contigs over the OLC algorism.

**Fig 3:**
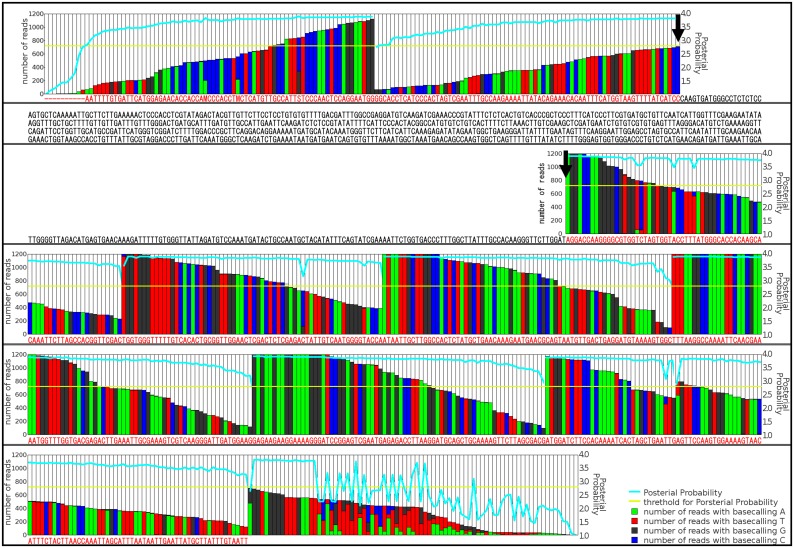
A typical example of the probability scores (blue line) and read counts (histogram) for VP-seq of *Forsythia* UGT7. The elongated sequence with VP-seq was indicated in red.

To validate the VP-seq method, we attempted to reconstitute three known sequences (CL1986Contig2 for FkUGT7, CL19667Contig1 for DXR and CL23194Contig2 for psd_Fi2), the full-length sequences of which failed to be assembled by Trinity. As shown in Fig 3 and S1–S3 Figs, the newly elongated sequences coincided with those deposited in the DDBJ database except for minor variants, probably due to the variations or polymorphisms within the species. In general, Trinity is considered to extend a contig, when various reads corresponding to the terminal regions of a contig are abundantly available. However, Trinity failed to extend contigs in the present study despite a high number of reads corresponding to the terminal regions of the contigs (Fig 3, arrows), suggesting that such incomplete assembly by Trinity resulted in the elucidation of only partial ORF sequences. Altogether, these results provide evidence that our VP-seq assembly method is the efficient and reliable method for assembling full-length ORFs from short Trinity-based contigs (input data 1) assembled by reads (input data 2) from the present *de novo* RNA-Seq.

**Fig 4:**
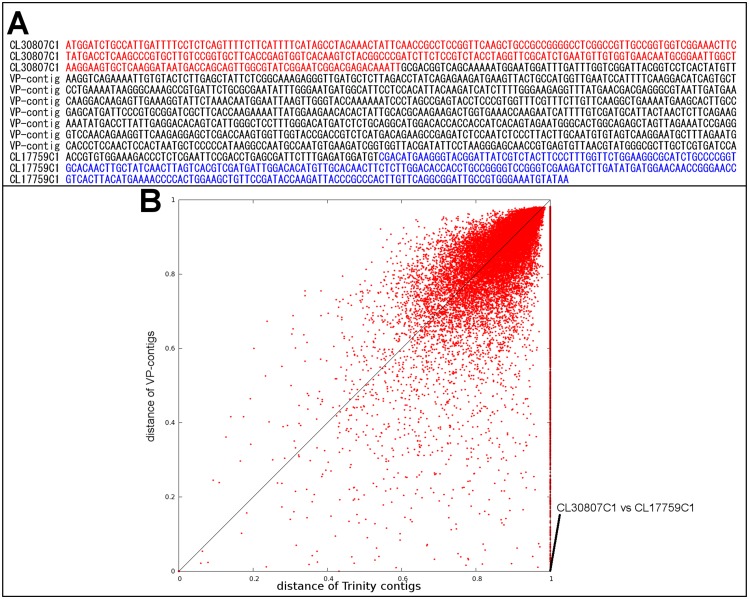
The sequence length (A) and FPKM value (B) distributions of Trinity- and virtual primer-based sequence assembly (VP-seq)-based contigs.

Subsequently, VP-seq of the aforementioned 1,576 contigs revealed that 480 contigs harbor redundant sequences. 709 of the independent 1,090 contigs were found to harbor full-length sequences (Table 3). A striking feature is that 563 new full-length ORF sequences were elucidated by VP-seq (Table 3). These data indicate that VP-seq improved the percentage of contigs containing a full-length ORF to 72%. Moreover, VP-seq was shown to elucidate the full-length ORF sequences of the contigs with a mean length of 1,146 base pairs via extension of 253 base pairs in average. As shown in Fig 4A, 68% of the VP-seq-based contigs were >1200 bp in length, whereas only 32% of the Trinity-based contigs were >1200 bp. Considering that the mean length of the reference UniProt enzyme sequences was 400 amino acids, we concluded that the length of the VP-seq-based contigs was sufficient for further annotation and molecular phylogenetic analyses, In addition, we examined the correlation between the depth (Fragment Per Kilobase Per Megareads, FPKM) and the percentage of full-length ORF contigs to total contigs elucidated by VP-seq (Fig 4B). Full-length ORF sequences for contigs with low expression levels (FPKM < 0.01) were not recovered, whereas those of 77% highly expressed contigs (FPKM >1) were elucidated (Fig 4B).

**Table 3:** Summary of the assembly of metabolism-related contigs from *Forsythia koreana* by Trinity or virtual primer-based sequence assembly (VP-seq).

	# contigs	Total length (bp)	Mean length (bp)	Full-length ORFs
Trinity	1576	1,348,038	855.35	146
VP-seq	1096	1,211,271	1145.95	709

ORF, open reading frame.

**Fig 5:**
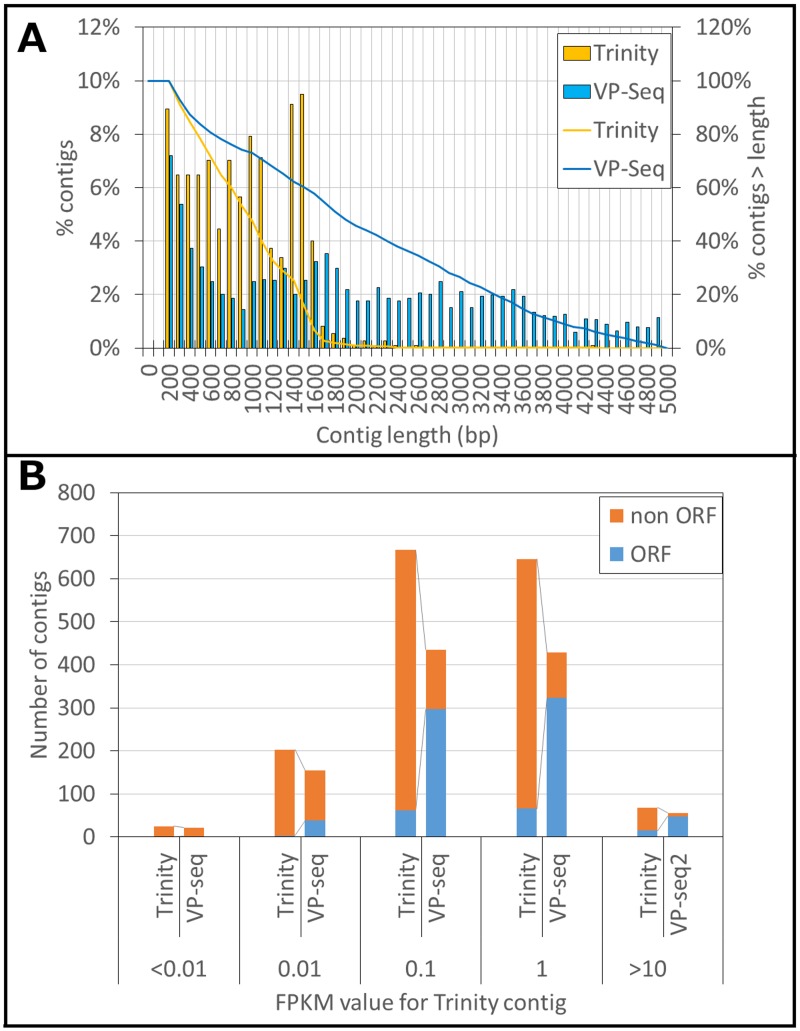
**(A) A typical example of distance-changed sequences.** Although no over-lapping region (distance = 1) was found between the Trinity-based contigs CL30807Contig1 (red) and CL177591Contig1 (blue), the two contigs were identical (distance = 0) after elongation by virtual primer-based sequence assembly (VP-seq). **(B) Relationship between the distances of Trinity-based and VP-seq-based contigs.**

We also compared the ORF sequences of the Trinity-based contigs and VP-seq-based contigs to determine factors causing sequence redundancy. The sequence alignment of these ORFs revealed that the redundancy was derived from 5’ and 3’fragments of the same transcripts (Fig 5A). Although such asymmetric alignments were observed in 10.6% of Trinity-based contig pairs, VP-seq reassembly reduced the percentage to 1.5% (Fig 5B). Since the asymmetric alignments among partial ORFs bias the estimation of copy numbers and phylogenetic distances of transcripts, full-length ORF sequences of contigs are requisite for the following quantification of expression levels and molecular phylogenetic analysis. In combination, these results confirmed the marked usefulness of the VP-Seq method for the elucidation of full-length ORF sequences of contigs.

### Structural classification of multiple metabolic enzyme genes

The large number of full-length ORF sequences obtained by VP-seq enabled a rigorous analysis of the distribution of contigs among Pfam-based structural classifications. The Pfam search identified 203 p450s, 82 UDP-glucose-dependent glucosyltransferases (UGTs), 62 non-heme dioxygenases, 54 transferases, 141 alcohol dehydrogenases, 9 Nmr A family members, 26 methyltransferases, and 13 chalcone and stilbene synthases. Of those 590 sequences, 34% (223) and 23% (137) were upregulated by more than two-fold in callus and leaf, respectively, which was much higher than the percentage of differentially expressed sequences across the whole transcriptome (3% for callus and 4% for leaf).

The GO:0009809 (lignin metabolism) includes lignan-biosynthetic genes. Thus, we further analyzed the VP-seq-based contigs against GO:000980, and verified the enrichment of 15 sequences in the callus library. The lignan pathway [26] (or see Fig 5) starts with deamination of phenylalanine by PAL1 to cinnamic acid, which is catalyzed to *p*-coumaric acid by CYP73A5. Then, *p*-coumaroyl-CoA is generated from *p*-coumaric acid by 4-coumarate-CoA ligases (4CLs: 4CL1, 4CL3, 4CLL6 and 4CLL7) and is converted to feruloyl-CoA via hydroxylation by FAH and HST, followed by methylation by F4IAT4. Feruloyl-CoA is catalyzed to coniferyl alcohol by cinnamoyl-CoA reductase I (CCR1) and cinnamyl alcohol dehydrogenases (CADs: CAD1, CAD5, CAD9 and ATCAD4).

**Fig 6:**
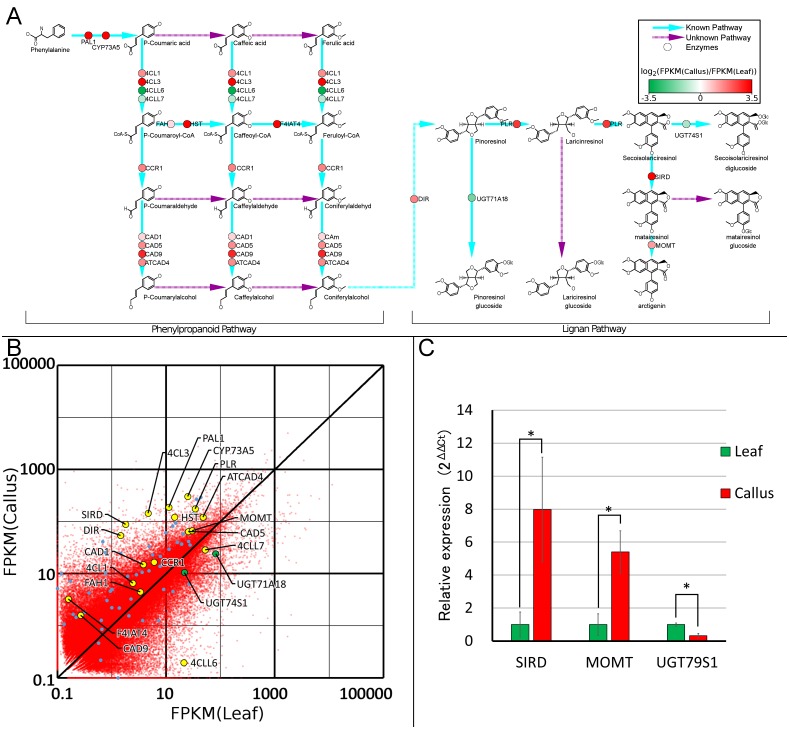
**(A) The FPKM distribution of contigs containing incomplete or full-length ORF sequences.** Identified (blue) and unidentified (purple) enzyme-catalyzed reactions. The color of the circles on the blue lines indicates the log ratio of the FPKM values of callus to leaf. FPKM, fragment per kilobase of transcript per million mapped reads; ORF, open reading frame. **(B) Expression of lignan-biosynthetic enzyme genes in callus and leaf.** Lignan-glucosylating UGTs, other lignan-biosynthetic enzymes and other plant specialized metabolism -related contigs are plotted in green, yellow and blue, respectively. **(C) Validation of RNA-Seq data by qRT-PCR.** qRT-PCR was performed for representative genes involved in biosynthesis downstream of lariciresinol, FkSIRD, FkMOMT and FkUGT74S1 that were shown to be differentially expressed by RNA-seq between *Forsythia* callus and leaf. Column charts and error bars indicate mean and standard errors of three biological replicates for each gene, respectively. Asterisks indicate a significant difference (Student’s s t-test; *P < 0.05).

Comparison of FPKMs between callus and leaf revealed that expression of the enzymes catalyzing the first two steps, *PAL1* and *CYP73A5*, were 16-fold and 12-fold upregulated in callus, respectively. The expression pattern of 4CLs varied among subtypes; *4CL1* and *4CL3* were 3-fold and 30-fold upregulated in callus, respectively, while *4CLL6* was 100-fold upregulated in leaf (Fig 6A and 6B). Since the FPKM value for *4CL3* in callus (144.3) was much larger than the value for *4CLL6* in leaf (28.7), 4-coumarate-CoA ligase activity is highly likely to be more potent in callus than leaf. The enzymes that convert *p*-coumaroyl-CoA to feruloyl-CoA (*FAH*, *HST* and *F4IAT4*) were also upregulated, although *FAH* and F4IAT4 were expressed at low levels (FPKM < 4) in both leaf and callus. The downstream reductases (*CAD1*, *5*, *9* and *ATCAD4*) and alcohol dehydrogenase were 2.6- to 2.8-fold upregulated in callus. Altogether, these analyses suggested that coniferyl alcohol, which is the direct precursor compound of a basal lignan, pinoresinol, was more abundantly synthesized in callus than in leaf.

The later lignan biosynthesis pathway originate from the coupling of stereo-specific coniferyl alcohol in the presence of diligent protein (DIR) [27], leading to the generation of pinoresinol. Pinoresinol is stepwisely reduced to lariciresinol and then secoisolariciresinol by pinoresinol-lariciresinol reductase (PLR). Secoisolariciresinol is converted into matairesinol by secoisolariciresinol dehydrogenase (SIRD). Furthermore, matairesinol is methoxylated to arctigenin by matairesinol *O*-methyltransferase (MOMT) [28]. These lignans are known to be glucosylated by UDP-glucose-dependent glucosyltransferases (UGTs). UGT71A18 [29], and UGT74S1 glucosylate pinoresinol and secoisolariciresinol [30] respectively, whereas no matairesinol- or arctigenin-glucosylating enzymes have been identified. All of the enzymes responsible for the biosynthesis of lignan aglycones were upregulated in callus. The expression levels of *DIR*, *PLR*, *SIRD*, and *MOMT* were 40-fold, 5-fold, 50-fold, and 2-fold higher in callus than in leaf, respectively (Fig 6A), proving that matairesinol biosynthesis is prominently enhanced and that matairesinol is highly accumulated in callus. Such intense expression of *SIRD* in callus is compatible with our previous findings that matairesinol, but not arctigenin, is specifically accumulated in *Forsythia* callus suspension culture [31], highlighting a novel pivotal role of matairesinol in the growth, proliferation, or differentiation of *Forsythia* callus. To confirm the results obtained by VP-Seq-based RNA-seq, the expression of three genes responsible for biosynthesis downstream of lariciresinol, FkSIRD, FkMOMT and FkUGT74S1 (Fig 6A), was examined by qRT-PCR, because these genes showed distinct callus-selective (FkSIRD and FkMOMT) or leaf-selective (FkUGT74S1) expression (Fig 6B). As shown in Fig 6C, FkSIRD and FkMOMT was found to be expressed predominantly in callus, whereas expression of FkUGT74S1 was much more intense in leaf than in callus. These results were in good agreement with the VP-seq-based RNA-seq data (Fig 6B), confirming the quality and reliability of VP-seq.

**Fig 7:**
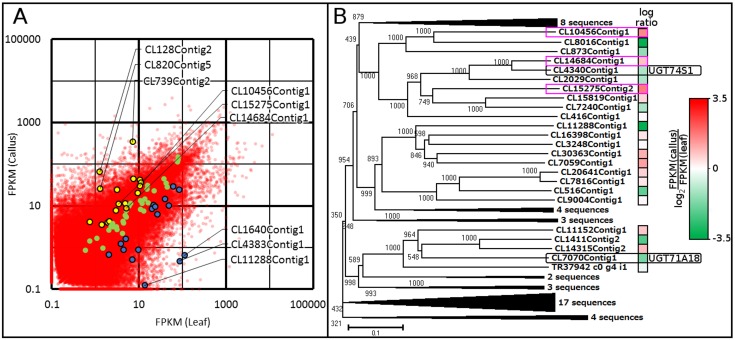
**(A) Gene expression in callus and leaf.** Contigs upregulated in callus, contigs upregulated in leaf, and contigs with no differential expression are plotted in yellow, blue and green, respectively. **(B) Phylogenetic tree of UGT-like sequences.** The color of the boxes beside each contig indicates the log ratios of the FPKM values of callus and leaf. FPKM, fragment per kilobase of transcript per million mapped reads.

Since lignans often accumulate as water-soluble glucosides in lignan producing plants, we also analyzed the expression levels of UGTs or their candidates, which were deduced from VP-seq-based contigs (Fig 7). *CL128Contig2* showed the most remarkable (52-fold) upregulation in *Forsythia* callus, although no known homologous UGTs were found by sequence comparison. To annotate the upregulated UGT genes in *Forsythia* callus, the molecular phylogenetic trees for UGT-like sequences of the full-length contigs were constructed using clustal W2 (Fig 7). It is noteworthy that the similarity of chemical structures of substrates for UGTs is implicated with the phylogenetic proximity of UGTs (Fig 7). Consistent with similar chemical structures of secoisolariciresinol and matairesinol (Fig 5), these data suggest that secoisolariciresinol glucosyltransferases (UGT74S1) is positioned proximally to a matairesinol glucosyltransferase that has not ever been identified. Notably, CL10456C1, CL14684Contig1 and CL15275Contig1 are also located at the proximal position to UGT74S1 (Fig 6B), although their substrates have yet to be identified. Moreover, CL10456Contig1, CL14684Contig1 and CL15275Contig1 exhibited 50% sequence identities to UGT74S1 (S1 Table), and the three glucosyltransferases were upregulated in callus by 2.9-, 1.7-, and 3.3-fold, respectively (Fig 6A and 6B). In keeping with the highest expression of SIRD (matairesinol biosynthetic enzyme) in callus as stated above, a matairesinol glucosyltransferase is highly likely to be upregulated in callus as well, given that a large portion of matairesinol was shown to be glucosylated in *Forsythia* callus suspension [31]. In combination, the molecular phylogenetic analyses of VP-seq-based full-length ORF sequences suggest that either some or all of the three *Forsythia* UGTs, CL10456Contig1, CL14684Contig1 and CL15275Contig1, are responsible for the glucosylation of matairesinol.

### VP-seq of other lignan-producing plant transcriptomes

Other lignan-rich plants biosynsethize structurally diverged lignans with beneficial biological effects on human, including anti-tumor activity of podophyllotoxin, which are not produced in *Forsythia* [4, 5], and RNA-seq data have been reported for several typical lignan-producing plants, including *L*. *flavum*, *L*. *usitatissimum*, and *P*. *hexandrum* [27, 32–34] (Table 4). The Illumina 54-bp paired end read data for other plants (*Linum flavum*, *Linum usitatissimum* and *Podophyllum hexandrum*) were downloaded from the MedPlantRNASeq DB [http://www.medplantrnaseq.org/] (Table 4). Since the RNA-seq analyses for these plants provide shorter reads than those of the present study (probably due to 54bp paired end reads), we used VP-seq to reassemble the data into longer contigs (Table 5).

**Table 4:** Metrics of the *de novo* assembly of the *Linum flavum*, *Linum usitatissimum*, and *Podophyllum hexandrum* transcriptomes.

	Sample	Read length	# reads	# contigs	N50 (bp)	Mean length (bp)	# PSM enzyme-related sequences
*L*. *flavum*	Root	54 bp × 2	18,680,786	78,475	1,666	1,034.0	1,867
*L*. *usitatissimum*	Seed coat	54 bp × 2	9,346,897	78,323	1,174	632.5	1,025
*P*. *hexandrum*	Rhizome	54 bp × 2	18,110,041	227,885	826	419.4	2,794

PSM, plant specialized metabolite.

**Table 5:** Summary of the assembly of metabolism-related contigs from *Linum flavum*, *Linum usitatissimum*, and *Podophyllum hexandrum* using Trinity (MedPlant DB) or virtual primer-based sequence assembly (VP-seq).

		# contigs	Total length (bp)	Mean length (bp)	Full-length ORFs
*L*. *flavum*	MedPlant DB ^a^	1,909	2,429,578	1,272.70	385
	VP-seq	965	1,138,305	1,179.59	812
*L*. *usitatissimum*	MedPlant DB	1,173	1,086,298	926.09	227
	VP-seq	527	541,787	1,028.06	401
*P*. *hexandrum*	MedPlant DB	2,146	1,960,346	913.49	286
	VP-seq	1,116	1,248,064	1,118.34	570

^a^ Trinity-based RNA-seq contigs were obtained from the MedPlant RNA Seq Database (http://www.medplantrnaseq.org; assembled contigs version 20101112).

A total of 1,909, 1,173 and 2,146 plant specialized metabolism -related contigs were collected for *L*. *flavum*, *L*. *usitatissimum*, and *P*. *hexandrum*, respectively. 80–90% of these contigs were shown to harbor incomplete ORFs, as detected in *Forsythia* (Table 5). Nevertheless, subsequent VP-Seq re-assembly for the contigs led to the elucidation of 427, 174 and 284 additional full-length ORFs for *L*. *flavum*, *L*. *usitatissimum* and *P*. *hexandrum*, respectively (Table 5). In addition, as in *Forsythia*, 45–55% contigs were found to be redundant sequences. Finally, we detected 812, 401, and 570 full-length ORFs in *L*. *flavum*, *L*. *usitatissimum* and *P*. *hexandrum*, respectively (Table 5). These also data confirmed the universal ability of VP-seq to reassemble reads as short as 50 bp.

**Fig 8:**
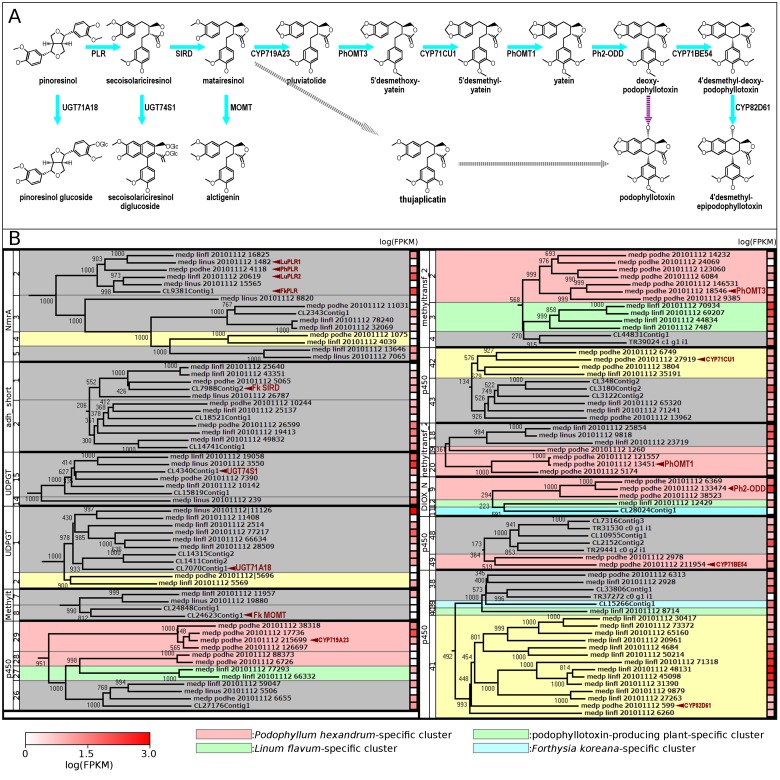
**(A) Biosynthesis pathways of lignans.** Identified (blue) or unidentified (gray and purple) enzyme-catalyzed reactions were indicated by solid and dash arrows, respectively. **(B) Phylogenetic trees for lignan biosynthesis-related enzymes.** *Podophyllum hexandrum*-, *Linum flavum*-, podophyllotoxin-producing plants-, and *Forsythia koreana*-specific clusters are highlighted in red, green, yellow, and blue, respectively. The color of the squares beside each contig indicates expression levels based on FPKM values of the corresponding genes.

To investigate the phylogenetic distribution of specialized metabolite enzyme genes, we performed comparative analysis among four lignan-rich plant species: *F*. *koreana*, *L*. *flavum*, *L*. *usitatissimum*, and *P*. *hexandrum*. The sequences harboring each of the 14 Pfam motifs were aligned with amino acid sequences in a Gonnet series, and phylogenetic trees were reconstructed using UPGMA with 1000 bootstrap replicates (S4–S17 Figs). Subsequently, we examined the relationship between the molecular phylogenetic distance of orthologs/ paralogs and the species-specificity of metabolic pathways, given that the four plant species possess unique lignan biosynthetic pathways. Analysis of the 14 resultant phylogenetic trees based on R function, cuttree, resulted in the generation of 353 gene clusters (Table 6). Of those, 32 clusters were shared by all of the four plant species. 58 and 71 clusters were shared with three and two species, respectively. Among the remaining 192 clusters, 52 contained two or more genes from a single species. Subsequently, we examined the molecular phylogenetic distance for orthologues/paralogs and the species-specificity of lignan biosynthesis-related genes, given that the four plant species possess unique lignan biosynthetic pathways downstream of secoisolariciresinol (Fig 8A). PLRs were shown to be present in all of the four plants (Fig 8B), which is compatible with the findings that pinoresinol, lariciresinol, and secoisolariciresinol are detected in these plants [3, 4]. Matairesinol, which is biosynthesized from secoisolariciresinol, has been detected in *F*. *koreana*, *L*. *flavum*, and *P*. *hexandrum*, and has been shown to undergo two metabolic pathways. First, *Forsythia* MOMT methylate matairesinol, leading to arctigenin [28], and second, a P450 family enzyme in *P*. *hexandrum*, CYP719A23, converts matairesinol to pluviatolide [34]. A recent study also demonstrated that pluviatolide was converted to yatein by three *P*. *hexandrum* enzymes: PhOMT3, CYP71CU1 and PhOMT1 [35] (Fig 8A). Although the key enzyme has yet to be fully identified, yatein is predicted to be the intermediate of podophyllotoxin, and Lau et. al. reported that three *P*. *hexandrum* enzymes participate in the biosynthesis of podophyllotoxin (Fig 8A). Additionally, thujaplicatin has been reported as an alternative intermediate in *Anthriscus sylvestris* [28]. Although intermediates and the enzymes involved in the conversion from matairesinol to podophyllotoxin have not yet been identified, podophyllotoxin has also been detected in *L*. *flavum* [3, 4].

**Table 6:** Summary of clusters generated by cuttree.

	Singleton				One specie				Two specie						Three specie				All	total
	Fk	Lf	Lu	Ph	Fk	Lf	Lu	Ph	Fk	Fk	Fk	Lf	Lf	Lu	Fk	Fk	Fk	Lf	Fk	
									Lf	Lu	Ph	Lu	Ph	Ph	Lf	Lf	Lu	Lu	Lf	
															Lu	Ph	Ph	Ph	Lu	
																			Ph	
ADH_N	3	0	3	4	1	0	0	0	1	0	2	2	0	0	0	0	2	7	3	28
ADH_zinc_N	3	1	2	3	0	0	0	1	0	1	1	2	0	0	0	0	1	4	3	22
Chal_sti_synt_N	2	1	0	3	1	1	0	0	1	1	1	2	0	1	0	1	0	1	1	17
DIOX_N	5	3	4	3	1	4	0	1	4	0	1	2	0	1	0	3	0	5	5	42
Methyltransf_11	1	1	1	0	0	0	0	0	0	1	0	2	0	0	1	1	0	1	2	11
Methyltransf_2	0	0	0	4	3	4	0	4	0	1	0	3	0	1	0	0	0	0	0	20
Methyltransf_3	4	2	0	2	0	0	0	1	0	1	1	0	1	0	0	0	0	0	1	13
NmrA	0	0	1	0	0	0	0	0	0	0	0	1	1	0	0	1	0	0	2	6
PPO1_DWL	0	1	0	1	1	1	0	0	0	0	0	2	0	0	0	0	0	0	0	6
Polyketide_cyc2	0	0	1	0	0	0	0	0	1	1	0	0	0	0	0	0	0	0	1	4
Transferase	12	7	6	3	2	4	0	2	0	1	1	3	0	1	2	1	0	0	4	49
UDPGT	4	1	5	5	1	1	0	3	1	0	2	1	1	0	3	0	2	1	1	32
adh_short	5	3	5	7	1	0	0	1	1	2	2	0	0	0	4	1	1	3	6	42
p450	8	4	4	2	4	4	1	4	2	1	2	2	8	0	4	3	3	2	3	61
Total	47	24	32	37	15	19	1	17	11	10	13	22	11	4	14	11	9	24	32	353

Fk, *Forsythia koreana*; Lf, *Linum flavum*; Lu, *Linum usitatissimum*; Ph, *Podophyllum hexandrum*

During our phylogenetic analysis, all known PLRs (FkPLR, LuPLR1, LuPLR2 and PhPLR) were clustered in NmrA-2 (Fig 8B). Although no PLR have yet been characterized in *L*. *flavum*, the NmrA-2 cluster also contained two *L*. *flavum* genes (medp_linfl_20101112_1685 and medp_linfl_20101112_20619), which are homologous to two *LuPLR*s (*LuPLR1* and *LuPLR2*). Furthermore, *medp_linfl_20101112_1685* and *medp_linfl_20101112_20619* were proximal to LuPLR1 and LuPLR2, respectively. In addition, the phylogenetic cluster indicated that *Linum PLR1s* and *PLR2s* were the most similar to *PhPLR* and *FkPLR*, respectively (Fig 8B). *LuPLR1*, *medp_linfl_20101112_1685* (*LfPLR1*), and *PhPLR* were expressed with FPKMs of 54.8, 20.6, and 25.2, respectively, whereas *FkPLR*, *LuPLR2*, and *medp_linfl_20101112_20619* (*LfPLR2*) were more highly expressed with FPKMs of 176.9, 220.7, and 124.0, respectively. The adh_short-1 cluster contained total 5 sequences, including FkSIRD, from the four species. Only *L*. *flavum* was shown to possess two paralogs (medp_linfl_20101112_25640 and medp_linfl_20101112_43351) in the cluster, both of which were significantly expressed (FPKM = 21.2 and 77.2, respectively). Together, these results suggest that the two SIRD-like genes are *L*. *flavum*-specific subtypes. *FkSIRD*, *PhSIRD*, and *medp_linus_20101112_26787* were expressed with FPKMs of 62.5, 11.8, and 62.5, respectively, confirming their biological significance (Fig 8B).

Pinoresinol and secoisolariciresinol are glucosylated by UGT71A18 and UGT74S1, respectively [29, 30]. UGT71A18 and UGT74S1 were included in the cluster UDPGT-13 and UDPGT-1, respectively. Both of the clusters are constituted by the genes from all four species. Unlike the UDPGT-1 cluster, the UDPGT-13 cluster was further separated into plant species-specific sub-clusters. For example, FkUGT74S1 formed a sub-cluster with two *F*. *koreana* UGTs (CL1411Contig2 and CL14315Contig2), and sub-cluster formation was also observed among the *L*. *flavum* UGTs (medp_linfl_201112_2514, medp_linfl_201112_77217, medp_linfl_201112_66634, and medp_linfl_20101112_28509). These molecular phylogenetic trees suggest the plant lineage-specific multiplication and diversification of UGT71A subfamily-related genes. FkMOMT, which converts matairesinol to arctigenin, was included in the *F*. *koreana*-specific cluster (cluster methyltransf_2–8), which coincides with the specific detection of arctigenin in *F*. *koreana* among the four plant species [3]. In contrast, the genes involved in the metabolic pathway from matairesinol to podophyllotoxin were restricted to podophyllotoxin-producing plant (*L*. *flavum* and *P*. *hexandrum*)-specific clusters, which is also in good agreement with the exclusive detection of podophyllotoxin in *L*. *flavum* and *P*. *hexandrum*.

CYP719A23, which converts matairesinol to pluviatolide [34], was located in the *P*. *hexandrum*-specific cluster, p450-29, and was expressed with the highest FPKM of 331.4 in the cluster (Fig 8B). In contrast, no CYP719A23-related *L*. *flavum*-specific clusters with FPKM values of > 10 were found. The lack of a CYP719A23-like contig in *L*. *flavum* suggests that the alternative podophyllotoxin metabolic pathway originated from convergent evolution. PhOMT3, which oxidizes pluviatolide to5’ desmethoxyyatein, was also located in the *P*. *hexandrum*-specific cluster, methyltransf_2–2, and was expressed with an FPKM of 10.4. Two other genes in the methyltransf_2–2 cluster, medp_podhe_20101112_9385 and medp_podhe_20101112_6084, exhibited high FPKM values of 20.6 and 19.0, respectively. Although a pluviatolide synthase (a CYP719A23-like gene) was not detected in the *L*. *flavum* transcriptome in the present study, the presence of the gene proximal to PhOMT3 suggested the existence of a similar catalytic pathway in *L*. *flavum*. In contrast to the two aforementioned enzymes, CYP71CU1, which oxidizes 5’ desmethoxyyatein to 5’ desmethylyatein [35], formed a podophyllotoxin-producing plants-specific cluster, p450-42 that contained the proximal *L*. *flavum* gene, *medp_linfl_20101112_3519*1. CYP71CU1 and medp_linfl_20101112_35191 exhibited the highest FPKMs in the cluster, with values of 20.8 and 18.2, respectively. Although little is known about the biosynthesis of podophyllotoxin in *L*. *flavum*, the structural similarity of medp_linfl_20101112_35191 to CYP71CU1 and high expression in lignan-producing plants suggests the involvement of this *F*. *flavum* P450 in lignan biosynthesis. Moreover, other enzymes for podophyllotoxin biosynthesis, PhOMT1 and Ph2-ODD, also formed *P*. *hexandrum*-specific clusters, Methyltransf_2–2 and DIOX_N-9, respectively. In each cluster, PhOMT1 and Ph2-ODD also exhibited the highest FPKMs with values of 16.3 and 272.4, respectively. Collectively, the phylogenetic clustering analyses support the notion that podophyllotoxin biosynthesis in the two species had evolved independently. Lau et al. also reported that two different P450 enzymes, CYP71BE54 and CYP82D61 are responsible for converting deoxypodophyllotoxin to 4’-desmethyl-deoxypodophyllotoxin and 4’-desmethyl-deoxypodophyllotoxin to 4’-desmthyl-epipodophyllotoxin, respectively [35]. CYP82D61 was clustered with 13 uncharacterized *L*. *flavum* P450s (cluster p450-41), *medp_linfl_20101112_48131* exhibited an outstanding FPKM value of 786.9 (ten-fold higher than that of CYP82D61). In contrast, no apparent CYP71BE54-related gene was found in transcriptomes from *L*. *flavum*, also underpinning the convergent evolution of podophyllotoxin biosynthetic enzymes.

We also identified ten *F*. *koreana*-specific clusters with FPKM values of > 10 (Table 7). Blast analysis indicated that five of the ten clusters are involved in terpenoid metabolic pathways. These results are compatible with a previous study demonstrating the identification of 3*β*-acetoxy-20*α*-hydroxyursan-28-oic acid, *β*-amyrin acetate, taraxasterol acetate, 3*β*-acetyl-20,25-epoxy-dammarane-24*α*-ol, acetyl oleanolic acid, betulinic acid, labda-8(17),13E-dien-15,18-dioic acid 15-methyl ester, and other tri- and di-terpenes specifically in *F*. *suspensa* [36, 37]. Particularly, 3*β*-acetoxy-20*α*-hydroxyursan-28-oic acid was a newly identified compound in *Forsythia* [36]. Taken together, these results indicate that the five clusters of genes participate in the *Forsythia*-specific terpenoid metabolic pathway.

**Table 7:** *Forsythia koreana*-specific clusters and cluster annotation from Blast2GO.

Cluster name	Annotation from Blast2GO	Potential pathway	Contigs	FPKM
p450.47	premnaspirodiene oxygenase-like	Monoterpene	CL7316Contig3	5.8
			TR31530_c0_g1_i1	17.7
			CL10955Contig1	5.2
			CL2152Contig2	124.7
			TR29441_c0_g2_i1	3.4
p450.02	CYP72A219-like	Monoterpene	CL120Contig6	3.9
			CL120Contig2	26.6
p450.60	ferruginol synthase-like	Diterpene	CL1762Contig1	96.6
			CL1762Contig2	119.1
adh_short.06	(-)-isopiperitenol (-)-carveol dehydrogenase, mitochondrial-like	Monoterpene	CL36802Contig1	0.2
			CL17092Contig1	3.7
			CL14923Contig2	110.1
p450.24	beta-amyrin 28-oxidase-like	Triterpene	CL27766Contig1	36.1
			CL16051Contig1	17.2
Transferase.13	spermidine hydroxycinnamoyl transferase-like		CL3486Contig3	19.9
			CL3486Contig1	64.0
Methyltransf_2.15	caffeic acid O-methyltransferase like		TR49393_c0_g2_i2	3.7
			CL167Contig12	42.5
Methyltransf_2.04	Uncharacterized methyltransferase		CL44831Contig1	4.1
			TR39024_c1_g1_i1	33.1
UDPGT.12	UDP-glucosyltransferase 86A1-like		CL1Contig598	8.3
			CL2128Contig4	25.4
			CL30763Contig1	3.5
Transferase.18	malonyl-coenzyme: anthocyanin 5-O-glucoside-6 -O-malonyltransferase-like		CL629Contig5	5.7
			CL5723Contig2	16.1
			CL29374Contig1	1.2

FPKM, fragment per kilobase of transcript per million mapped reads.

## Conclusion

We have successfully developed a novel bioinformatics application, VP-seq, which enables effective elucidation of full-length ORF sequences via extension of incomplete contigs generated by Trinity *de novo* assembly of *F*. *koreana* leaf and callus. Furthermore, we demonstrated comparative VP-seq-based transcriptome analyses of major lignan-producing plants leading to the detection of complete ORF sequences of candidate genes for unidentified lignan biosynthesis pathways. Thus, VP-seq accelerated the characterization of enzymes that catalyze undetermined steps in lignan biosynthesis and is also widely applicable to the elucidation of full-length ORF sequences of any *de novo* assembled incomplete contigs in transcriptomes.

The molecular phylogenetic analysis of specialized metabolic enzyme genes from lignan-producing plants revealed both common gene clusters that include genes from various plants and plant lineage-specific gene clusters. The former include enzymes involved in the early common lignan biosynthesis upstream of matairesinol such as PLR and SIRD, suggesting that they have occurred in their ancestral plants and conserved their biological functions. In contrast, the latter, such as CYP82D61, are likely to participate in lignan biosynthesis downstream of matairesinol. The unique gene sets for podophyllotoxin biosynthesis in *P*. *hexandrum* and *L*. *flavum*, which were originally verified by rigorous analyses of VP-seq-based full-length ORFs, also provide insight into the molecular evolution and diversity of species-specific enzymes involved in biosynthesis of various structurally related lignans at later steps, and the molecular basis for novel transgenic metabolic engineering of these lignan-producing plants.

## Supporting Information

S1 Fig Alignment between VP-seq reconstituted contig CL1986Contig2 and FkUGT7. The elongated sequences with VP-seq was highlighted with yellow and variants with and without amino acid substitutions were indicated in red and blue, respectively.
(TIF)

S2 Fig Alignment between VP-seq reconstituted contig CL19667Contog1 and FkDXR. The elongated sequences with VP-seq was highlighted with yellow and variants with and without amino acid substitutions were indicated in red and blue, respectively.
(TIF)

S3 Fig Alignment between VP-seq reconstituted contig CL23194Contig2 and psd_Fi2. The elongated sequences with VP-seq was highlighted with yellow and variants with and without amino acid substitutions were indicated in red and blue, respectively.
(TIF)

S4 Fig Phylogenetic tree for enzymes harboring the Pfam domain, ADH_N. *Podophyllum hexandrum*-, *Linum flavum*-, podophyllotoxin-producing plants-, and *Forsythia koreana*-specific clusters are highlighted in red, green, yellow, and blue, respectively. The color of the squares beside each contig indicates expression levels based on FPKM values of the corresponding genes.
(TIF)

S5 Fig Phylogenetic tree for enzymes harboring the Pfam domain, adh_short. *Podophyllum hexandrum*-, *Linum flavum*-, podophyllotoxin-producing plants-, and *Forsythia koreana*-specific clusters are highlighted in red, green, yellow, and blue, respectively. The color of the squares beside each contig indicates expression levels based on FPKM values of the corresponding genes.
(TIF)

S6 Fig Phylogenetic tree for enzymes harboring the Pfam domain, ADH_zinc_N. *Podophyllum hexandrum*-, *Linum flavum*-, podophyllotoxin-producing plants-, and *Forsythia koreana*-specific clusters are highlighted in red, green, yellow, and blue, respectively. The color of the squares beside each contig indicates expression levels based on FPKM values of the corresponding genes.
(TIF)

S7 Fig Phylogenetic tree for enzymes harboring the Pfam domain, Chal_sti_synt_N. *Podophyllum hexandrum*-, *Linum flavum*-, podophyllotoxin-producing plants-, and *Forsythia koreana*-specific clusters are highlighted in red, green, yellow, and blue, respectively. The color of the squares beside each contig indicates expression levels based on FPKM values of the corresponding genes.
(TIF)

S8 Fig Phylogenetic tree for enzymes harboring the Pfam domain, DIOX_N. *Podophyllum hexandrum*-, *Linum flavum*-, podophyllotoxin-producing plants-, and *Forsythia koreana*-specific clusters are highlighted in red, green, yellow, and blue, respectively. The color of the squares beside each contig indicates expression levels based on FPKM values of the corresponding genes.
(TIF)

S9 Fig Phylogenetic tree for enzymes harboring the Pfam domain, Methyltransf_2. *Podophyllum hexandrum*-, *Linum flavum*-, podophyllotoxin-producing plants-, and *Forsythia koreana*-specific clusters are highlighted in red, green, yellow, and blue, respectively. The color of the squares beside each contig indicates expression levels based on FPKM values of the corresponding genes.
(TIF)

S10 Fig Phylogenetic tree for enzymes harboring the Pfam domain, Methyltransf_3. *Podophyllum hexandrum*-, *Linum flavum*-, podophyllotoxin-producing plants-, and *Forsythia koreana*-specific clusters are highlighted in red, green, yellow, and blue, respectively. The color of the squares beside each contig indicates expression levels based on FPKM values of the corresponding genes.
(TIF)

S11 Fig Phylogenetic tree for enzymes harboring the Pfam domain, Methyltransf_11. *Podophyllum hexandrum*-, *Linum flavum*-, podophyllotoxin-producing plants-, and *Forsythia koreana*-specific clusters are highlighted in red, green, yellow, and blue, respectively. The color of the squares beside each contig indicates expression levels based on FPKM values of the corresponding genes.
(TIF)

S12 Fig Phylogenetic tree for enzymes harboring the Pfam domain, NmrA. *Podophyllum hexandrum*-, *Linum flavum*-, podophyllotoxin-producing plants-, and *Forsythia koreana*-specific clusters are highlighted in red, green, yellow, and blue, respectively. The color of the squares beside each contig indicates expression levels based on FPKM values of the corresponding genes.
(TIF)

S13 Fig Phylogenetic tree for enzymes harboring the Pfam domain, p450. *Podophyllum hexandrum*-, *Linum flavum*-, podophyllotoxin-producing plants-, and *Forsythia koreana*-specific clusters are highlighted in red, green, yellow, and blue, respectively. The color of the squares beside each contig indicates expression levels based on FPKM values of the corresponding genes.
(TIF)

S14 Fig Phylogenetic tree for enzymes harboring the Pfam domain, Polyketide_cyc2. *Podophyllum hexandrum*-, *Linum flavum*-, podophyllotoxin-producing plants-, and *Forsythia koreana*-specific clusters are highlighted in red, green, yellow, and blue, respectively. The color of the squares beside each contig indicates expression levels based on FPKM values of the corresponding genes.
(TIF)

S15 Fig Phylogenetic tree for enzymes harboring the Pfam domain, PPO1_DWL. *Podophyllum hexandrum*-, *Linum flavum*-, podophyllotoxin-producing plants-, and *Forsythia koreana*-specific clusters are highlighted in red, green, yellow, and blue, respectively. The color of the squares beside each contig indicates expression levels based on FPKM values of the corresponding genes.
(TIF)

S16 Fig Phylogenetic tree for enzymes harboring the Pfam domain, Transferase. *Podophyllum hexandrum*-, *Linum flavum*-, podophyllotoxin-producing plants-, and *Forsythia koreana*-specific clusters are highlighted in red, green, yellow, and blue, respectively. The color of the squares beside each contig indicates expression levels based on FPKM values of the corresponding genes.
(TIF)

S17 Fig Phylogenetic tree for enzymes harboring the Pfam domain, UDPGT. *Podophyllum hexandrum*-, *Linum flavum*-, podophyllotoxin-producing plants-, and *Forsythia koreana*-specific clusters are highlighted in red, green, yellow, and blue, respectively. The color of the squares beside each contig indicates expression levels based on FPKM values of the corresponding genes.
(TIF)

S1 Table Sequence identity of VP-seq-based contigs to UGT74S1 and UGT71A18. (PDF)

## Abbreviations

FPKM: fragment per kilobase of transcript per million mapped reads
GO: gene ontology
MOMT: matairesinol *O*-methyltransferase
PLR: pinoresinol/lariciresinol reductase
RT-PCR: reverse transcription PCR
SIRD: secoisolariciresinol diglucoside
UPGMA: unweighted pair group method with arithmetic mean
UGT: UDP-glucose-dependent glucosyltransferases
VP-seq: virtual primer-based sequence assembly
