# Supplementary figures and images for: De Novo Transcriptomes of Forsythia koreana Using a Novel Assembly Method: Insight into Tissue- and Species-Specific Expression of Lignan Biosynthesis-Related Gene
Source: PLoS One. 2016 Oct 21;11(10):e0164805. doi: 10.1371/journal.pone.0164805 (PMC5074596; doi:10.1371/journal.pone.0164805)

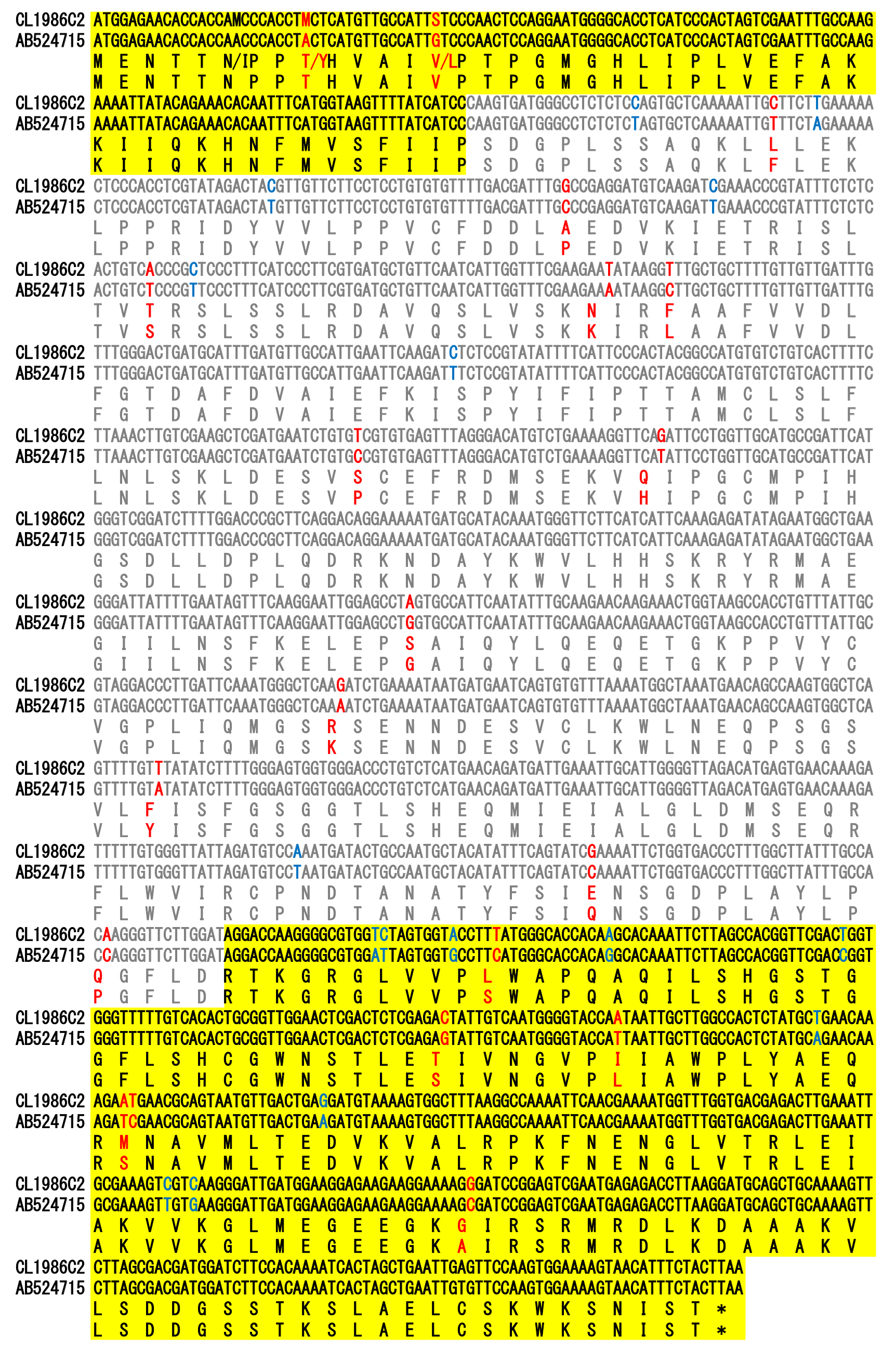

Supplement: S1 Fig — The elongated sequences with VP-seq was highlighted with yellow and variants with and without amino acid substitutions were indicated in red and blue, respectively. (TIF) [file pone.0164805.s001.tif]

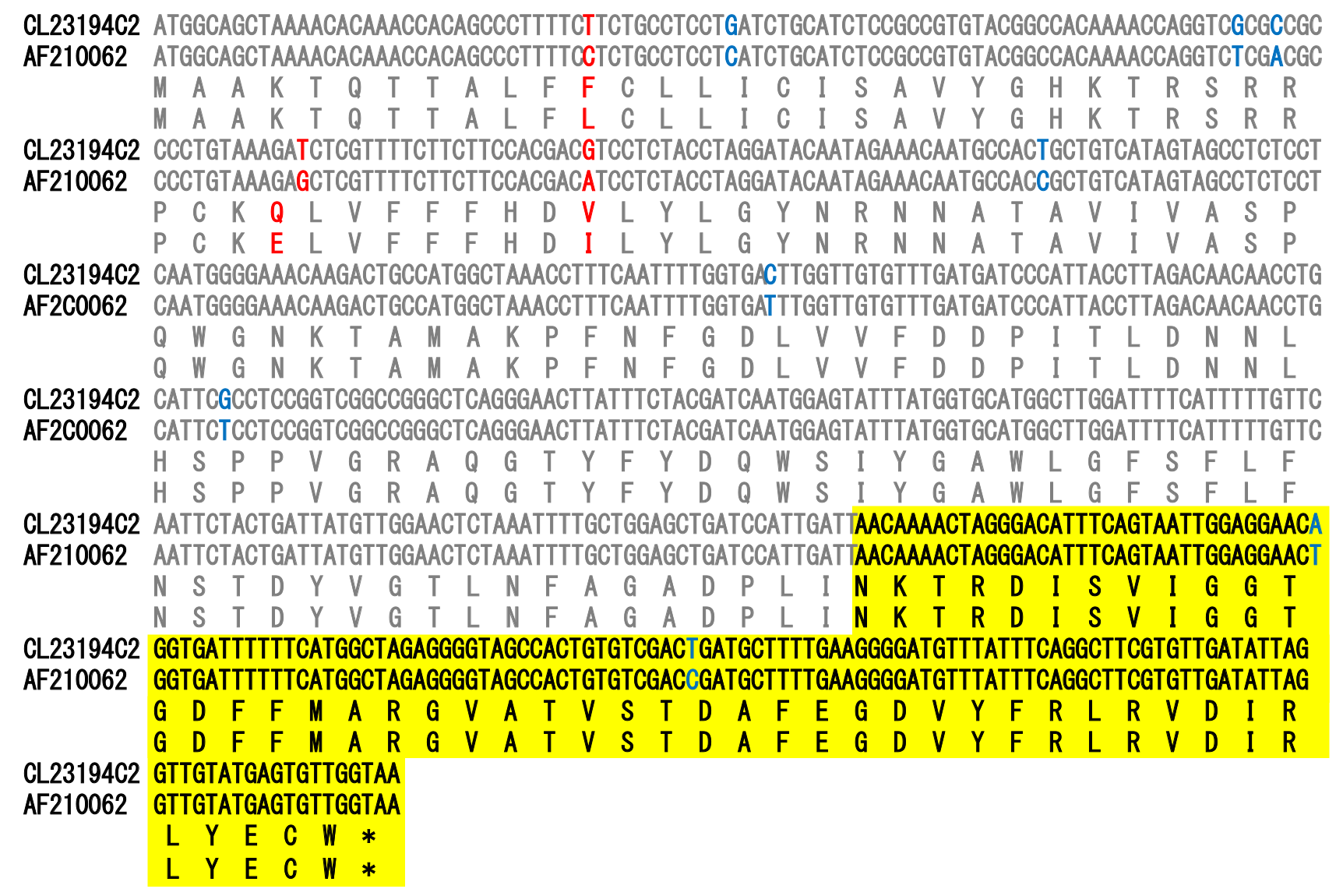

Supplement: S2 Fig — The elongated sequences with VP-seq was highlighted with yellow and variants with and without amino acid substitutions were indicated in red and blue, respectively. (TIF) [file pone.0164805.s002.tif]

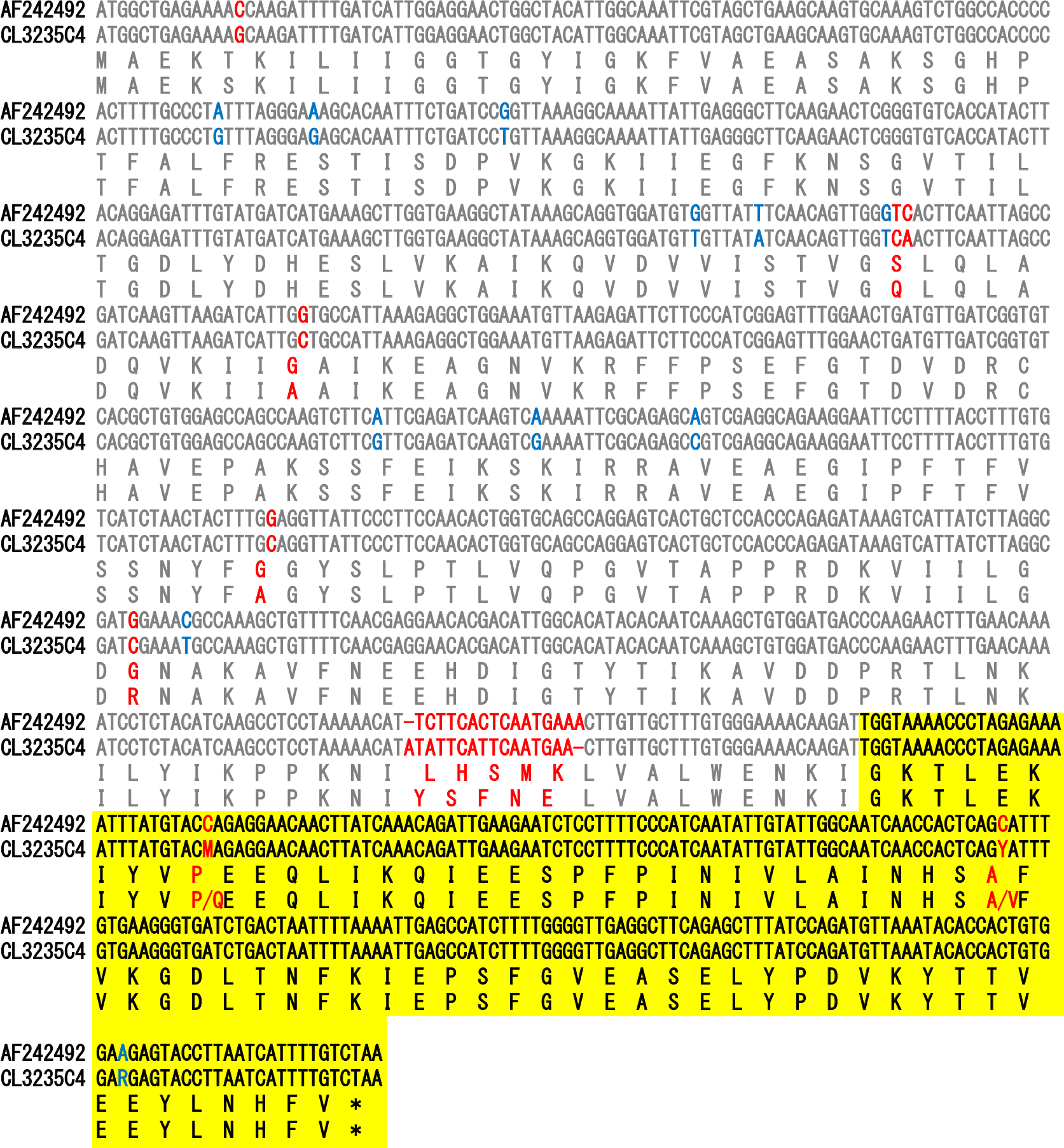

Supplement: S3 Fig — The elongated sequences with VP-seq was highlighted with yellow and variants with and without amino acid substitutions were indicated in red and blue, respectively. (TIF) [file pone.0164805.s003.tif]

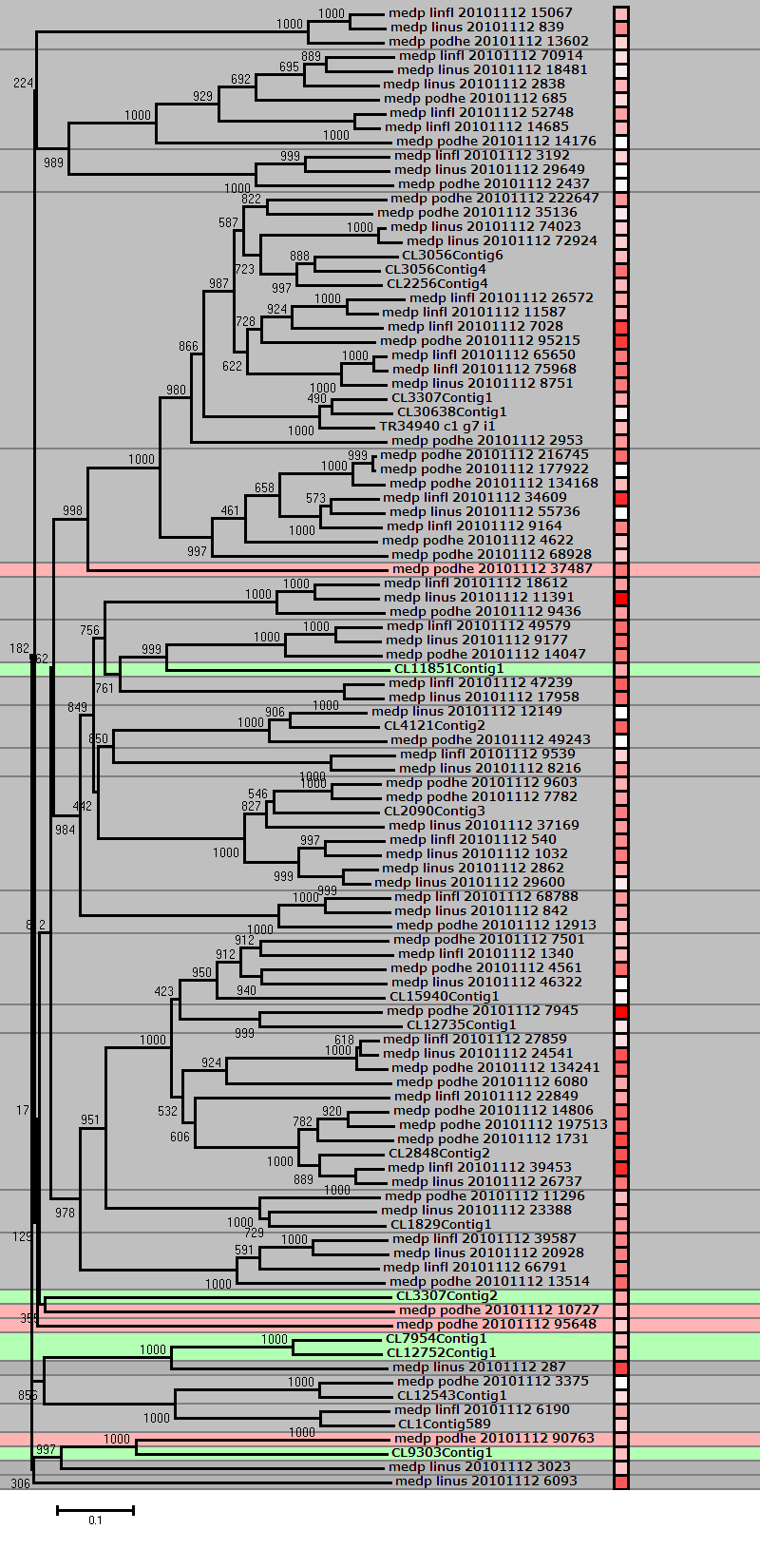

Supplement: S4 Fig — Podophyllum hexandrum-, Linum flavum-, podophyllotoxin-producing plants-, and Forsythia koreana-specific clusters are highlighted in red, green, yellow, and blue, respectively. The color of the squares beside each contig indicates expression levels based on FPKM values of the corresponding genes. (TIF) [file pone.0164805.s004.tif]

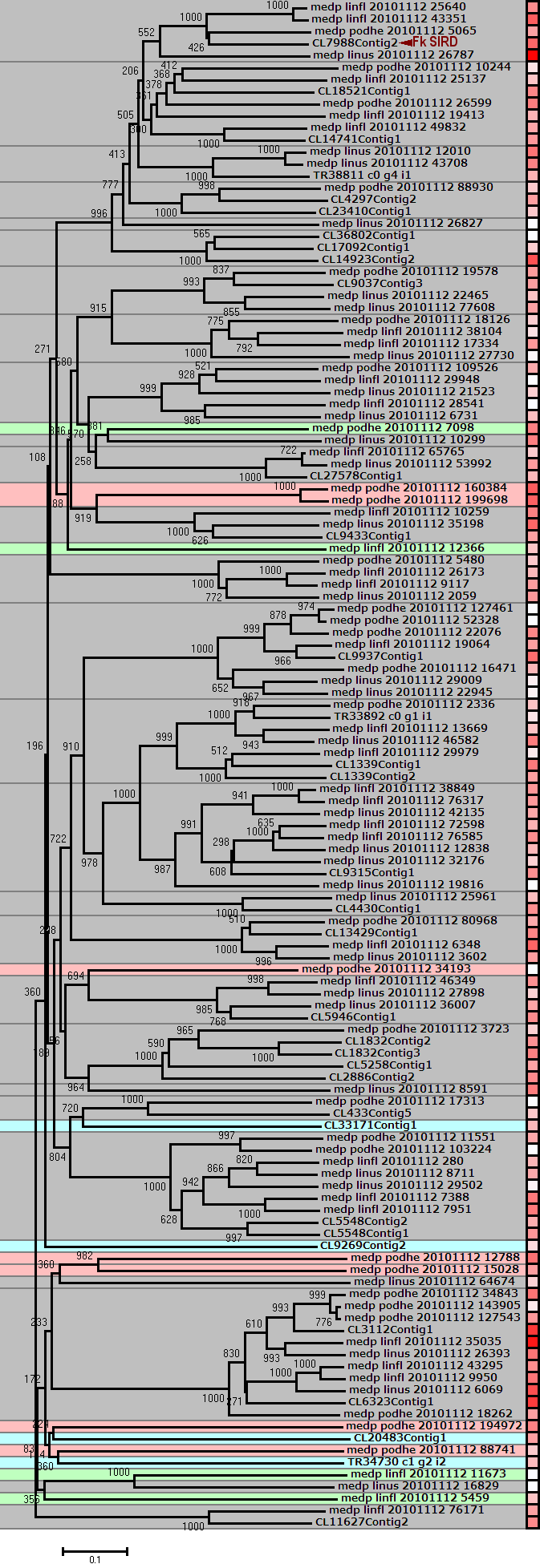

Supplement: S5 Fig — Podophyllum hexandrum-, Linum flavum-, podophyllotoxin-producing plants-, and Forsythia koreana-specific clusters are highlighted in red, green, yellow, and blue, respectively. The color of the squares beside each contig indicates expression levels based on FPKM values of the corresponding genes. (TIF) [file pone.0164805.s005.tif]

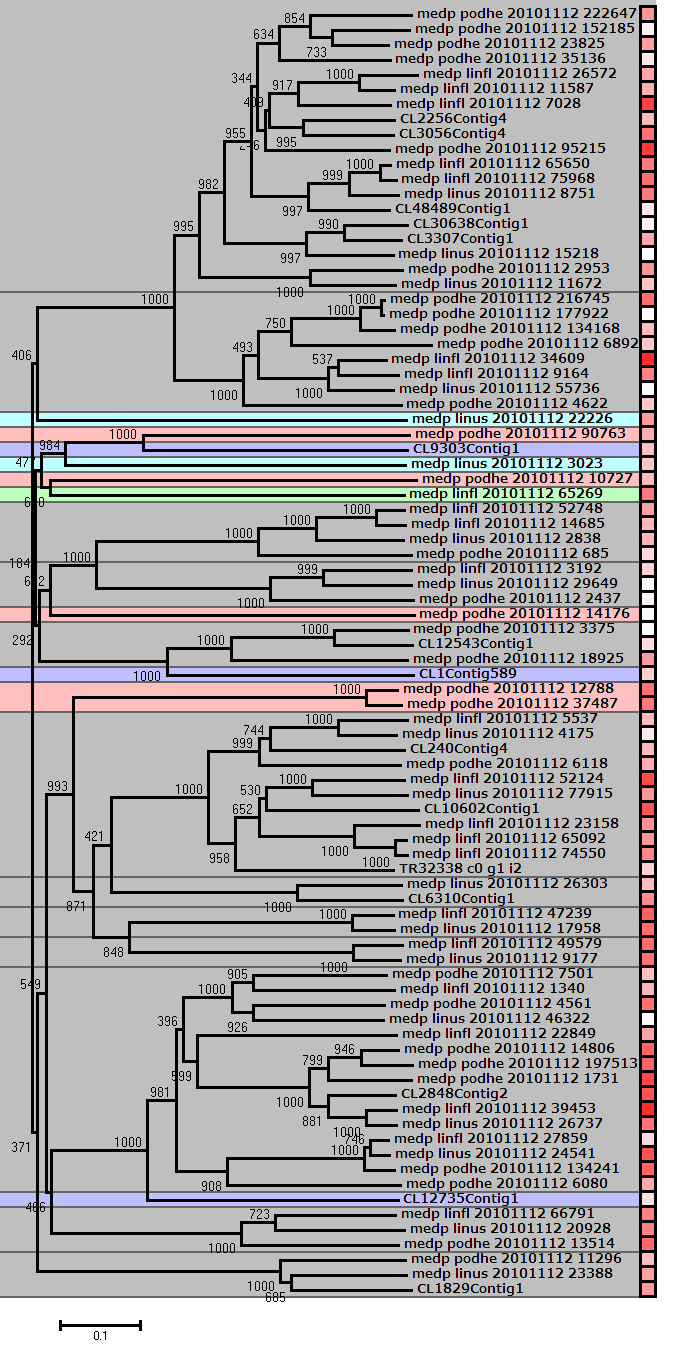

Supplement: S6 Fig — Podophyllum hexandrum-, Linum flavum-, podophyllotoxin-producing plants-, and Forsythia koreana-specific clusters are highlighted in red, green, yellow, and blue, respectively. The color of the squares beside each contig indicates expression levels based on FPKM values of the corresponding genes. (TIF) [file pone.0164805.s006.tif]

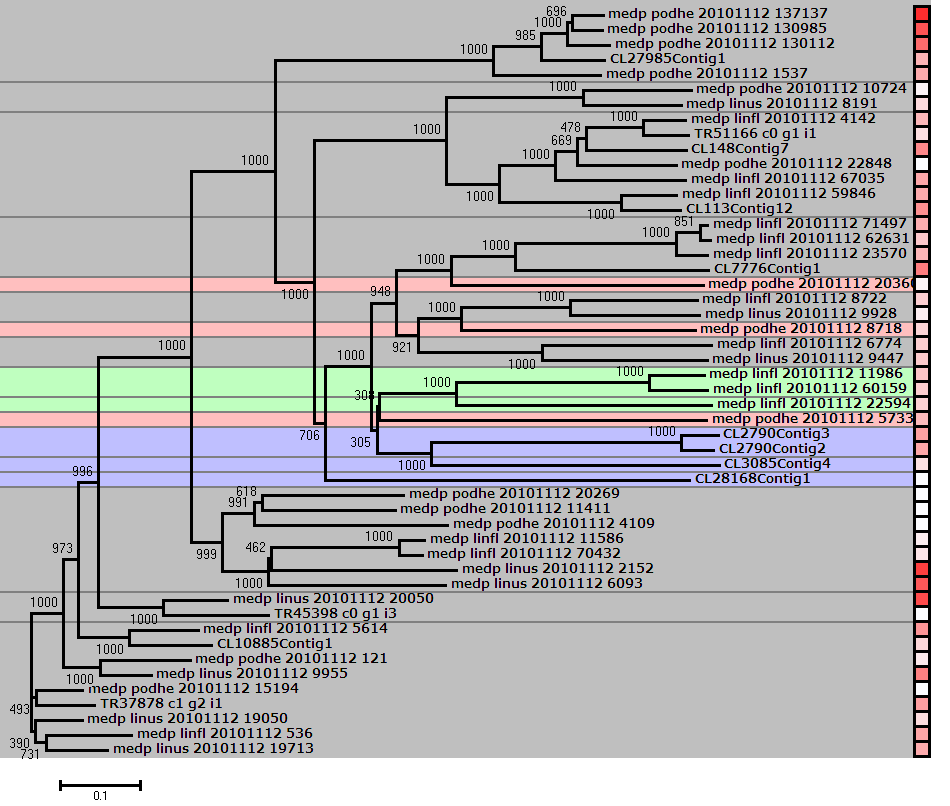

Supplement: S7 Fig — Podophyllum hexandrum-, Linum flavum-, podophyllotoxin-producing plants-, and Forsythia koreana-specific clusters are highlighted in red, green, yellow, and blue, respectively. The color of the squares beside each contig indicates expression levels based on FPKM values of the corresponding genes. (TIF) [file pone.0164805.s007.tif]

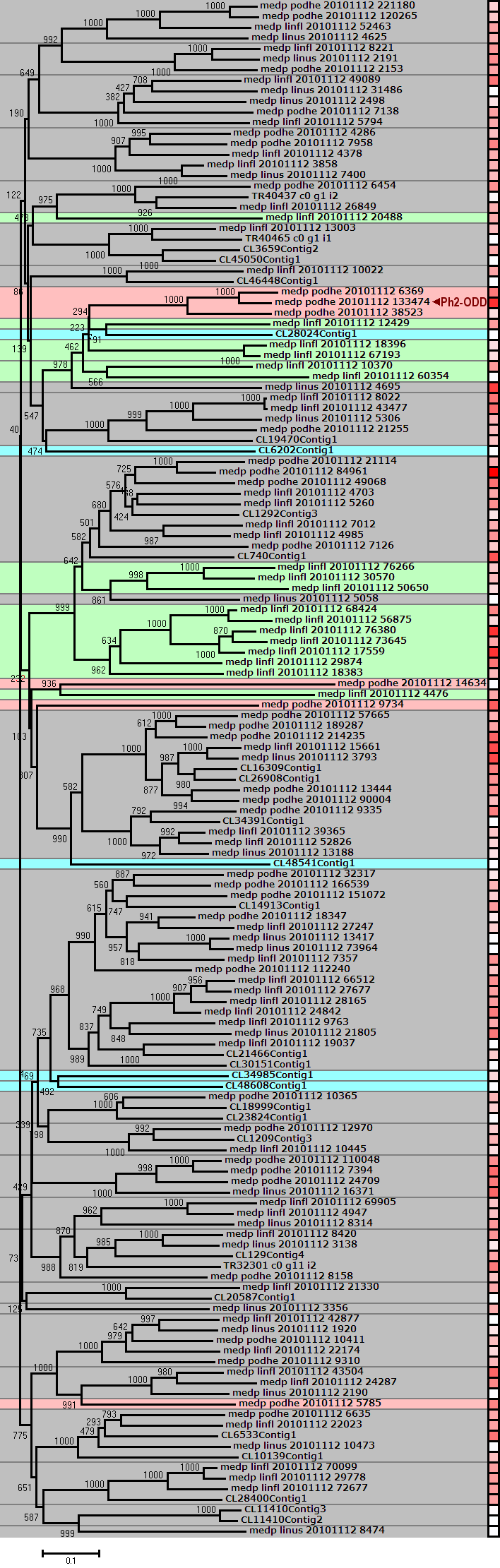

Supplement: S8 Fig — Podophyllum hexandrum-, Linum flavum-, podophyllotoxin-producing plants-, and Forsythia koreana-specific clusters are highlighted in red, green, yellow, and blue, respectively. The color of the squares beside each contig indicates expression levels based on FPKM values of the corresponding genes. (TIF) [file pone.0164805.s008.tif]

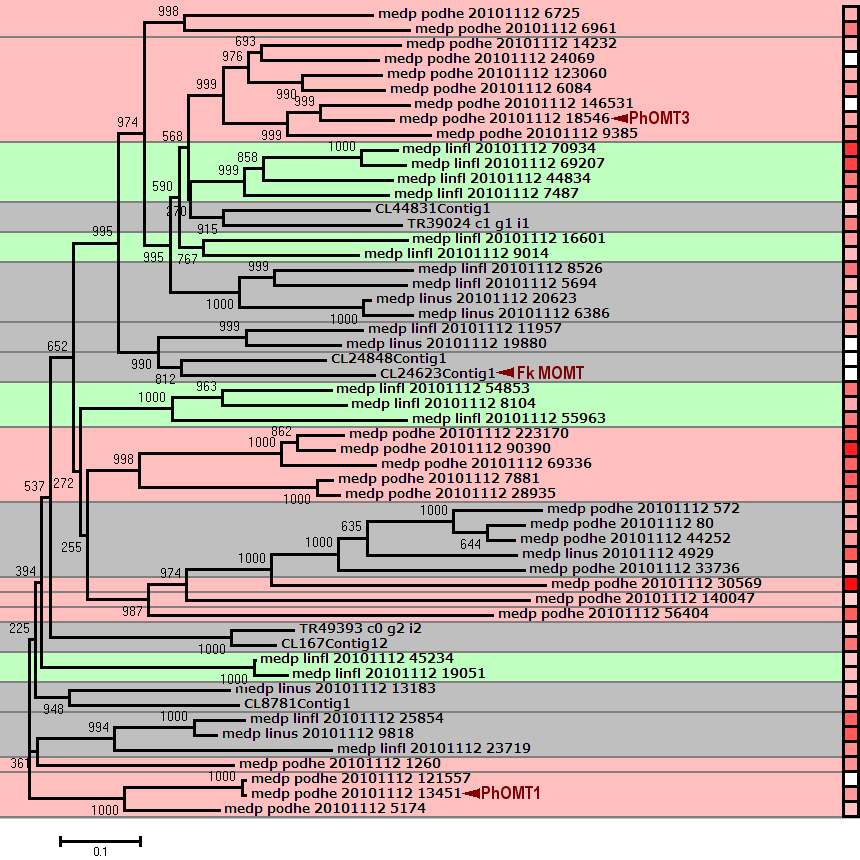

Supplement: S9 Fig — Podophyllum hexandrum-, Linum flavum-, podophyllotoxin-producing plants-, and Forsythia koreana-specific clusters are highlighted in red, green, yellow, and blue, respectively. The color of the squares beside each contig indicates expression levels based on FPKM values of the corresponding genes. (TIF) [file pone.0164805.s009.tif]

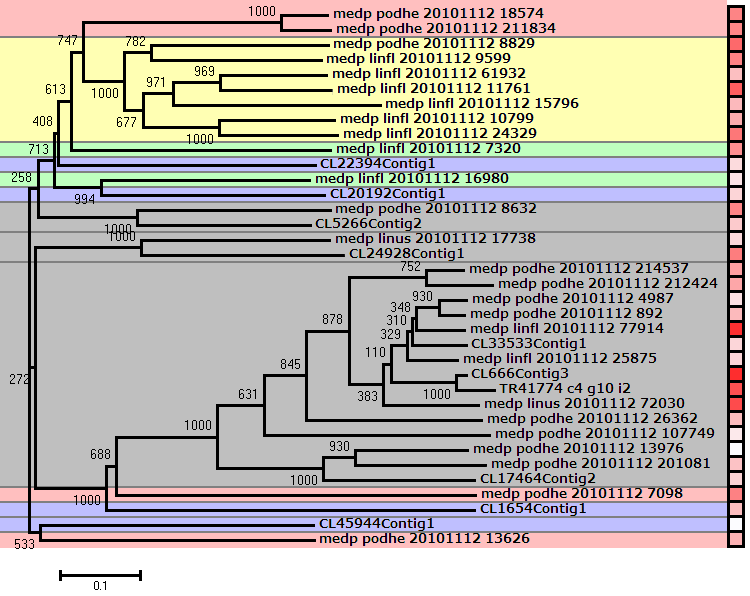

Supplement: S10 Fig — Podophyllum hexandrum-, Linum flavum-, podophyllotoxin-producing plants-, and Forsythia koreana-specific clusters are highlighted in red, green, yellow, and blue, respectively. The color of the squares beside each contig indicates expression levels based on FPKM values of the corresponding genes. (TIF) [file pone.0164805.s010.tif]

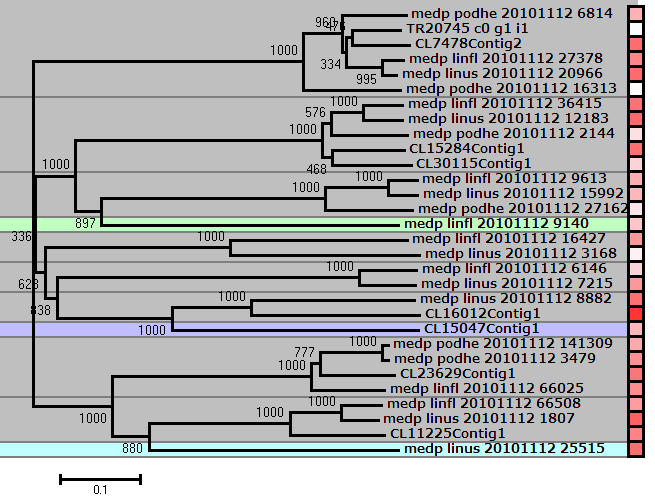

Supplement: S11 Fig — Podophyllum hexandrum-, Linum flavum-, podophyllotoxin-producing plants-, and Forsythia koreana-specific clusters are highlighted in red, green, yellow, and blue, respectively. The color of the squares beside each contig indicates expression levels based on FPKM values of the corresponding genes. (TIF) [file pone.0164805.s011.tif]

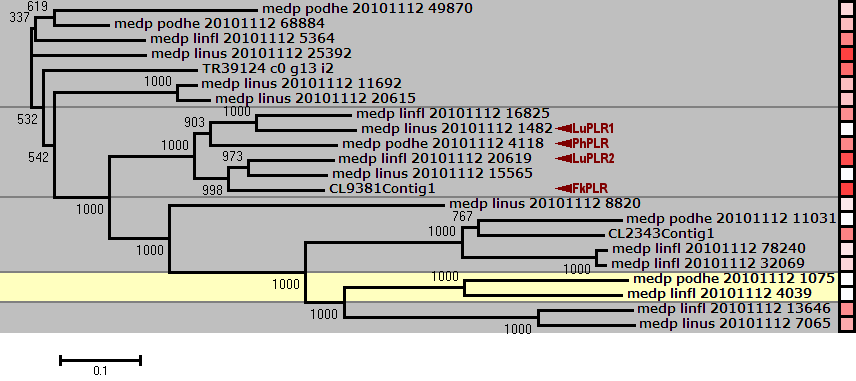

Supplement: S12 Fig — Podophyllum hexandrum-, Linum flavum-, podophyllotoxin-producing plants-, and Forsythia koreana-specific clusters are highlighted in red, green, yellow, and blue, respectively. The color of the squares beside each contig indicates expression levels based on FPKM values of the corresponding genes. (TIF) [file pone.0164805.s012.tif]

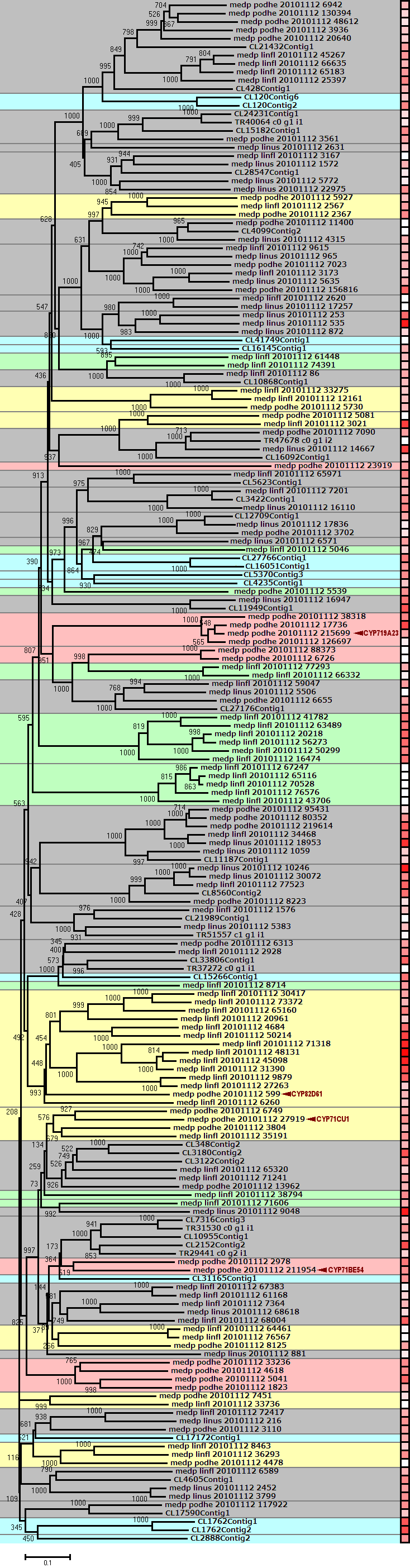

Supplement: S13 Fig — Podophyllum hexandrum-, Linum flavum-, podophyllotoxin-producing plants-, and Forsythia koreana-specific clusters are highlighted in red, green, yellow, and blue, respectively. The color of the squares beside each contig indicates expression levels based on FPKM values of the corresponding genes. (TIF) [file pone.0164805.s013.tif]

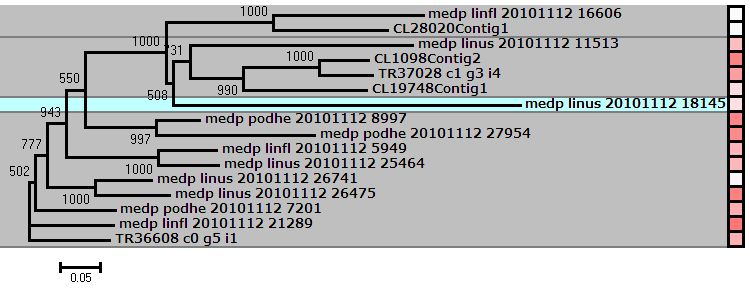

Supplement: S14 Fig — Podophyllum hexandrum-, Linum flavum-, podophyllotoxin-producing plants-, and Forsythia koreana-specific clusters are highlighted in red, green, yellow, and blue, respectively. The color of the squares beside each contig indicates expression levels based on FPKM values of the corresponding genes. (TIF) [file pone.0164805.s014.tif]

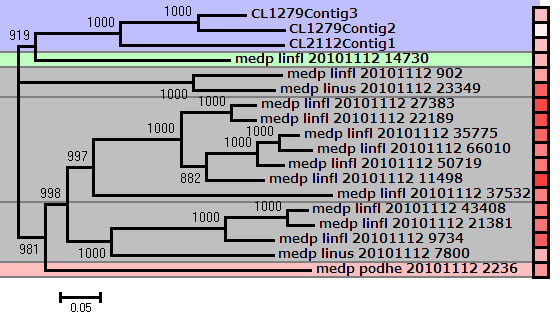

Supplement: S15 Fig — Podophyllum hexandrum-, Linum flavum-, podophyllotoxin-producing plants-, and Forsythia koreana-specific clusters are highlighted in red, green, yellow, and blue, respectively. The color of the squares beside each contig indicates expression levels based on FPKM values of the corresponding genes. (TIF) [file pone.0164805.s015.tif]

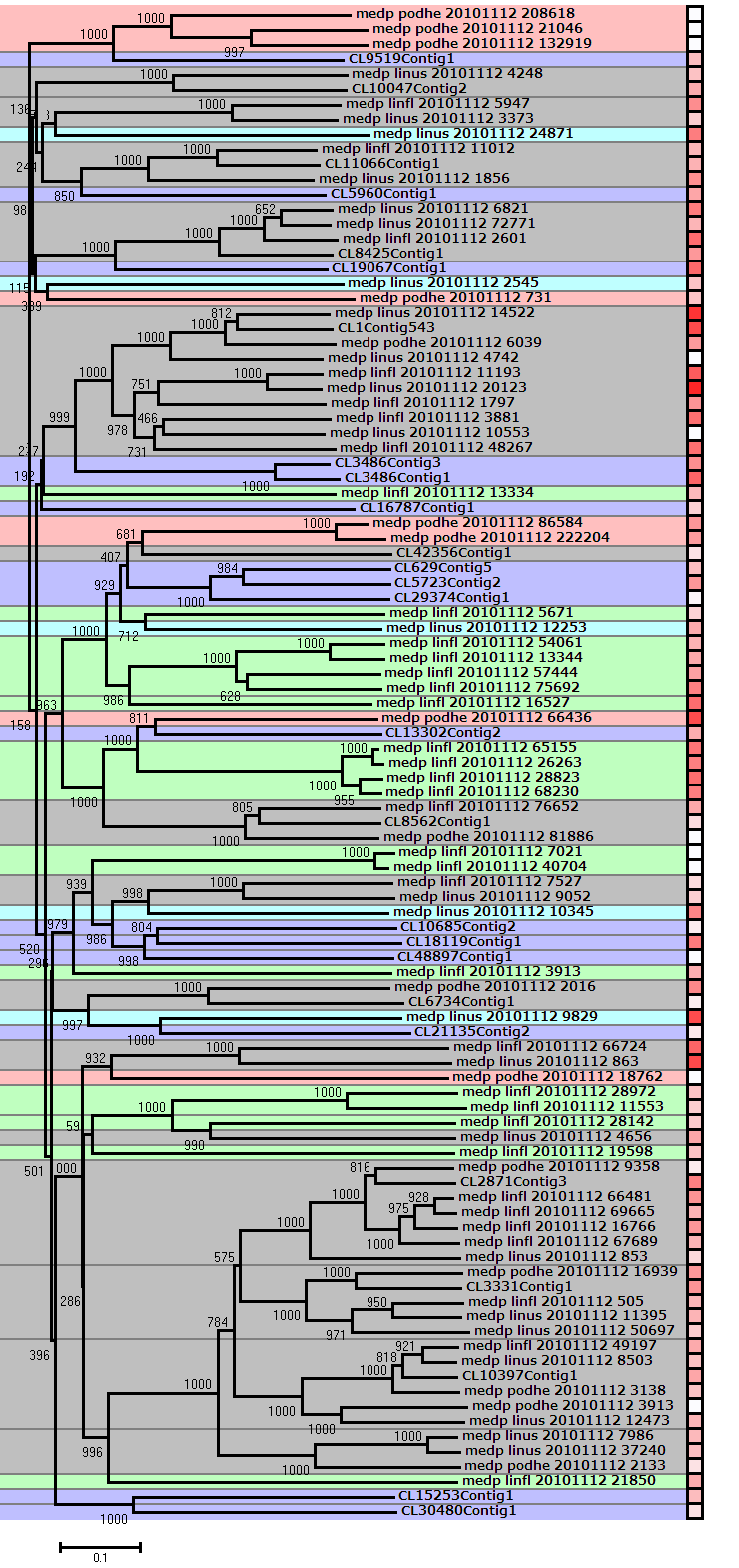

Supplement: S16 Fig — Podophyllum hexandrum-, Linum flavum-, podophyllotoxin-producing plants-, and Forsythia koreana-specific clusters are highlighted in red, green, yellow, and blue, respectively. The color of the squares beside each contig indicates expression levels based on FPKM values of the corresponding genes. (TIF) [file pone.0164805.s016.tif]

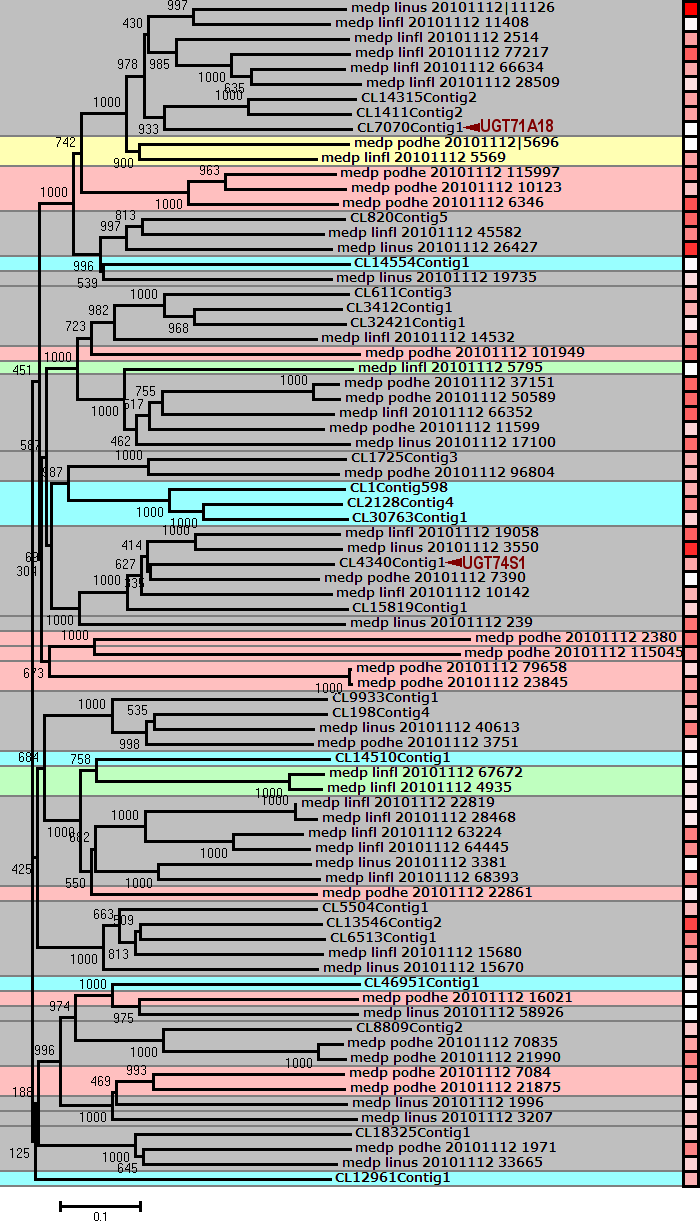

Supplement: S17 Fig — Podophyllum hexandrum-, Linum flavum-, podophyllotoxin-producing plants-, and Forsythia koreana-specific clusters are highlighted in red, green, yellow, and blue, respectively. The color of the squares beside each contig indicates expression levels based on FPKM values of the corresponding genes. (TIF) [file pone.0164805.s017.tif]
