# Supplementary material for: De Novo Transcriptomes of Forsythia koreana Using a Novel Assembly Method: Insight into Tissue- and Species-Specific Expression of Lignan Biosynthesis-Related Gene
Source: PLoS One. 2016 Oct 21;11(10):e0164805. doi: 10.1371/journal.pone.0164805 (PMC5074596; doi:10.1371/journal.pone.0164805)
Supplement: S1 Table — (PDF) [file pone.0164805.s018.pdf]

contig name	hit sequence		e-value	Identity with UGT74S1	Identity with UGT71A18
CL10456Contig1	AAX16493.1	UDP-glucose:flavonoid 7-O -glucosyltransferase [Ma...	0	36.4%	21.4%
CL8016Contig1	BAM28983.1	UDP-glucose crocetin gluco -syltransferase [Garden...	0	35.3%	22.5%
CL873Contig1	XP_007099701	UDP-glucose:flavonoid 7-O-glucosyltransferas...	1.00E-152	36.0%	23.3%
CL14684Contig1	NP_001274852	UDP-glycosyltransferase 74E2-like [Solanum t...	0	82.1%	22.1%
CL4340Contig1	EXB61153.1	UDP-glycosyltransferase 74F2 [Morus notabilis]	0	100.0%	22.0%
CL2029Contig1	EXB61153.1	UDP-glycosyltransferase 74F2 [Morus notabilis]	6.00E-176	64.2%	21.5%
CL15275Contig2	EXB88302.1	UDP-glycosyltransferase 74F2 [Morus notabilis]	0	53.8%	22.1%
CL15819Contig1	EXB88302.1	UDP-glycosyltransferase 74F2 [Morus notabilis]	5.00E-156	45.1%	21.6%
CL7240Contig1	EXB88302.1	UDP-glycosyltransferase 74F2 [Morus notabilis]	3.00E-154	46.6%	23.3%
CL416Contig1	ACM09993.3	UDP-glycosyltransferase BMGT1 [Bacopa monnieri]	2.00E-180	47.2%	22.6%
CL11288Contig1	XP_007042920	UDP-glucosyl transferase 85A2 [Theobroma cac...	0	28.4%	23.5%
CL16398Contig1	XP_007042921	UDP-glucosyl transferase 85A3 [Theobroma cac...	0	27.2%	24.0%
CL3248Contig1	BAO51833.1	UDP-glycosyltransferase 85K10 [Camellia sinensis]	0	25.9%	22.1%
CL30363Contig1	BAO51844.1	UDP-glycosyltransferase 85A28 [Vitis vinifera]	0	29.5%	25.6%
CL7059Contig1	F8WKW1.1	UGT2_GARJA 7-deoxy -loganetin glucosyltr...	0	30.7%	25.9%
CL20641Contig1	U3U992.1	UGT8_CATRO 7-deoxy -loganetic acid gluco...	0	26.7%	23.7%
CL7816Contig1	AGX93065.1	7-deoxyloganetic acid UDP -glucosyltransferase [Ca...	0	26.3%	23.4%
CL516Contig1	AGX93065.1	7-deoxyloganetic acid UDP -glucosyltransferase [Ca...	0	27.7%	25.0%
CL9004Contig1	U3U992.1	UGT8_CATRO 7-deoxy -loganetic acid gluco...	0	26.2%	24.1%
CL11152Contig1	BAF75901.1	tetrahydroxychalcone 2'-glucosyltransferase [Cat...	2.00E-164	22.1%	49.7%
CL1411Contig2	AFD61601.1	anthocyanidin 3-O-glucosyl -transferase [Hevea bras...	1.00E-166	23.7%	50.7%
CL14315Contig2	AFD61601.1	anthocyanidin 3-O-glucosyl -transferase [Hevea bras...	7.00E-159	24.0%	49.8%
CL7070Contig1	BAF75901.1	tetrahydroxychalcone 2'-glucosyltransferase [Cat...	0	22.0%	100.0%
TR37942_c0_g4_i1	Q2V6K0.1	UFOG6_FRAAN UDP -glucose flavonoid 3-O-...	4.00E-142	21.8%	44.4%
